# Supplementary material for: Emergence of SARS-CoV-2 subgenomic RNAs that enhance viral fitness and immune evasion
Source: PLoS Biol. 2025 Jan 21;23(1):e3002982. doi: 10.1371/journal.pbio.3002982 (PMC11774490; doi:10.1371/journal.pbio.3002982)
Supplement: S1 Fig — (A) Schematic for detection of N, N.ORF3 and ORF9b-specfic sgmRNA using the endpoint reverse transcription PCR (RT-PCR) assay depicted in Fig 2E–G. (B) Nanopore sequencing of endpoint PCR products from N.iORF3-, N-, or ORF9b-specific sgmRNAs in clinical swab samples from EU1 and Alpha lineages, expressed as either raw Nanopore read numbers, determined by TRS-B junction-specific sequences, (left panel) or expressed as a proportion of N reads per sample (right panel). Data are means and standard errors of 12 swab samples per lineage and p-values represent pairwise t-tests for each sgmRNA species. TRS, transcription regulatory sequence. Data underlying this figure can be found in: https://doi.org/10.25418/crick.27952842. (PDF) [file pbio.3002982.s001.pdf]

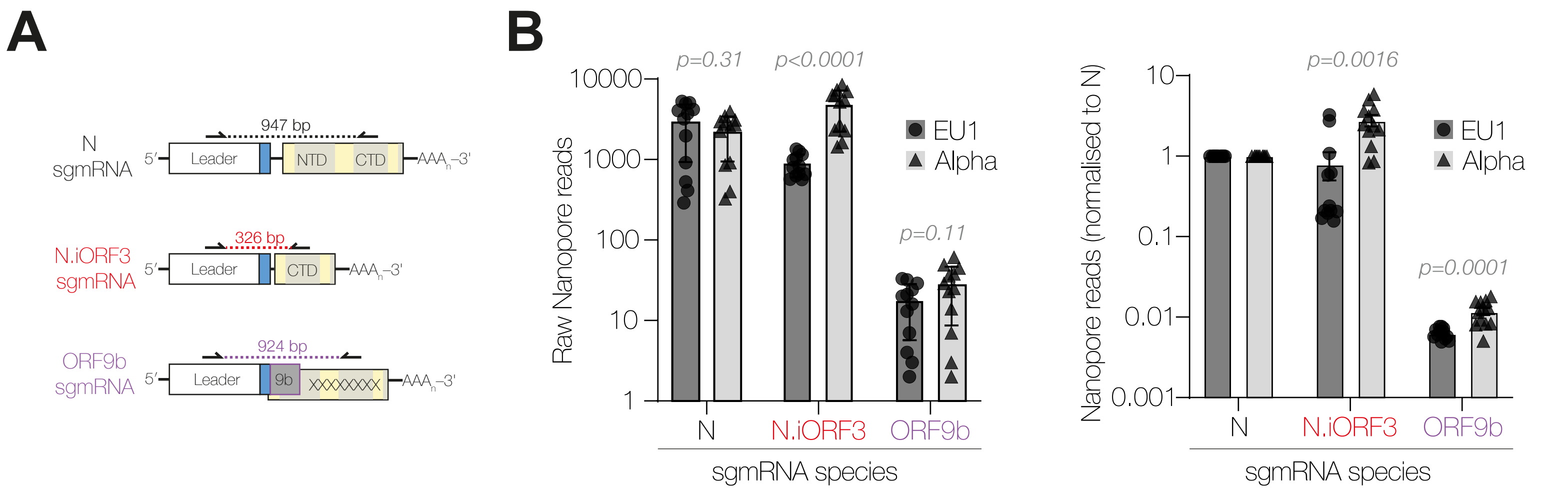

Fig. S1. Nanopore sequencing of sgmRNA amplicons from human swab samples. (**A**) Schematic for detection of N, N.ORF3 and ORF9b-specfic sgmRNA using the endpoint RT-PCR assay depicted in Fig. 2E-G. (**B**) Nanopore sequencing of endpoint PCR products from N.iORF3-, N-, or ORF9b-specific sgmRNAs in clinical swab samples from EU1 and Alpha lineages, expressed as either raw Nanopore read numbers, determined by TRS-B junction-specific sequences, (left panel) or expressed as a proportion of N reads per sample (right panel). Data are means and standard errors of twelve swab samples per lineage and p values represent pairwise t-tests for each sgmRNA species. Data underlying this figure can be found in: https://-doi.org/10.25418/crick.27952842.
