# Supplementary material for: Emergence of SARS-CoV-2 subgenomic RNAs that enhance viral fitness and immune evasion
Source: PLoS Biol. 2025 Jan 21;23(1):e3002982. doi: 10.1371/journal.pbio.3002982 (PMC11774490; doi:10.1371/journal.pbio.3002982)
Supplement: S2 Fig — (A) Phylogenetic reconstruction of the Iota variant evolution (B.1.526), highlighting the emergence of N.iORF3 (red). (B) A silent mutation at S202 generates extended homology of the N.iORF3 TRS-B region to the 5′UTR region flanking the TRS-L. (C) Phylogenetic reconstruction of SARS-CoV-2 evolution in humans, with independent emergences of extended N.iORF3 TRS sequence highlighted (see S1 Table). The phylogenetic tree in panel a was adapted from Nextstrain based on the Iota-focussed build, and in panel C on the Omicron.21K focussed-build [103,104]. (D) Proportion of SARS-CoV-2 sequences with the extended N.iORF3 TRS-B sequence by lineage, showing B.1.1 and P.1 (Gamma) in the left panel, and Alpha and four Omicron sub-lineages in the right panel. (E) Venn diagram showing proportion of B.1.1 sequences (red) with the A28877U, G28878C mutations (blue), and minimal emergence of these mutations outside of the B.1.1 lineage. TRS, transcription regulatory sequence. Data underlying this figure can be found in: https://doi.org/10.25418/crick.27952842. (PDF) [file pbio.3002982.s002.pdf]

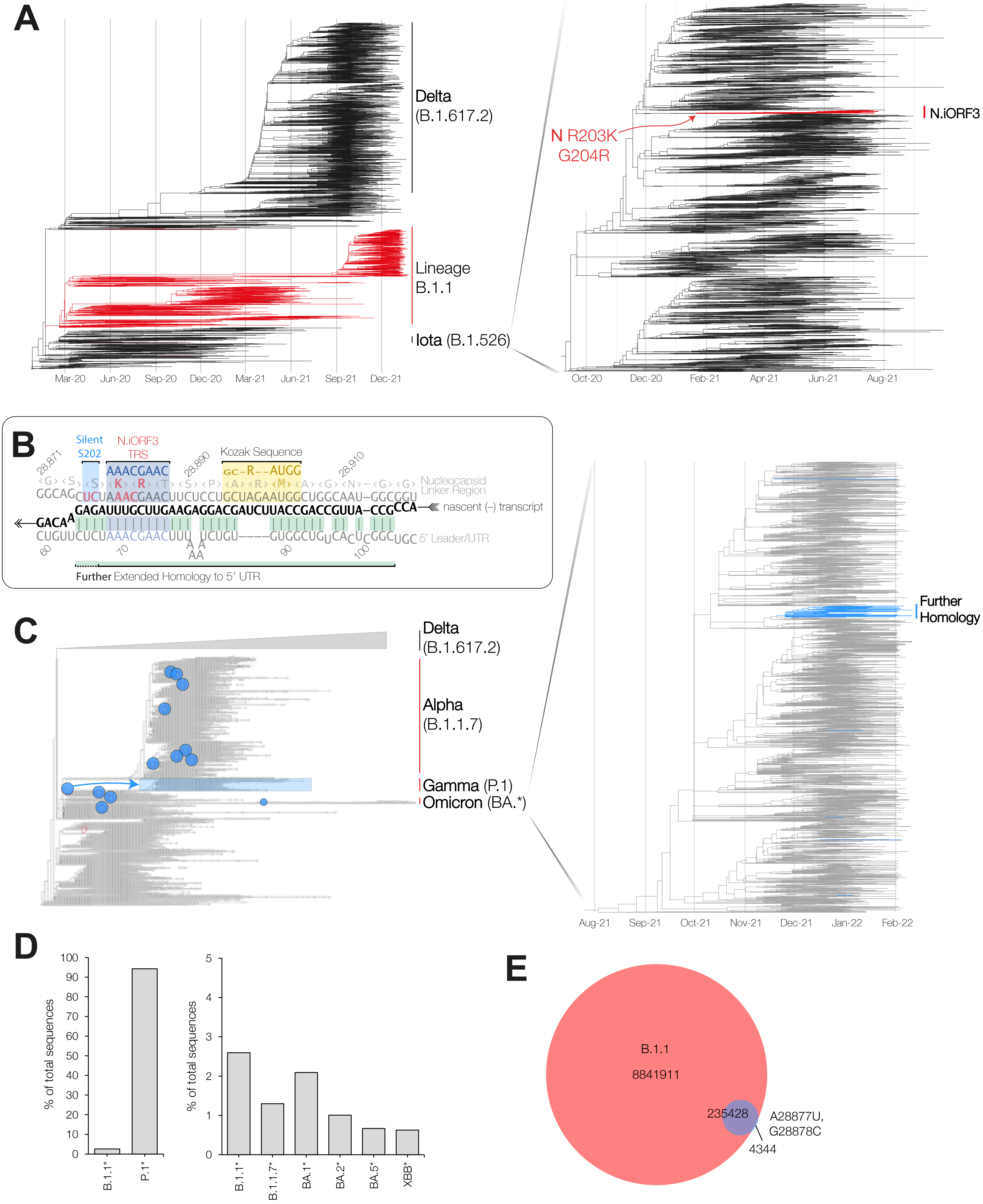

Fig. S2. N.iORF3 and extended homology to TRS-flanking regions have both evolved convergently. (**A**) Phylogenetic reconstruction of the Iota variant evolution (B.1.526), highlighting the emergence of N.iORF3 (red). (**B**) A silent mutation at S202 generates extended homology of the N.iORF3 TRS-B region to the 5′UTR region flanking the TRS-L. (**C**) Phylogenetic reconstruction of SARS-CoV-2 evolution in humans, with independent emergences of extended N.iORF3 TRS sequence highlighted (See **Table S1**). The phylogenetic tree in panel a was adapted from Nextstrain based on the Iota-focused build, and in panel c on the Omicron.21K focused-build (78, 79). (**D**) Proportion of SARS-CoV-2 sequences with the extended N.iORF3 TRS-B sequence by lineage, showing B.1.1 and P.1 (Gamma) in the left panel, and Alpha and four Omicron sub-lineages in the right panel. (**E**) Venn diagram showing proportion of B.1.1 sequences (red) with the A28877U, G28878C mutations (blue), and minimal emergence of these mutations outside of the B.1.1 lineage. Data underlying this figure can be found in: https://doi.org/10.25418/crick.27952842.
