# Supplementary material for: Emergence of SARS-CoV-2 subgenomic RNAs that enhance viral fitness and immune evasion
Source: PLoS Biol. 2025 Jan 21;23(1):e3002982. doi: 10.1371/journal.pbio.3002982 (PMC11774490; doi:10.1371/journal.pbio.3002982)
Supplement: S3 Fig — (A) Schematic of the SARS-CoV-2 genome (upper panel) and frequency of emergence of the TRS-B sequence (AAACGAAC) in the global SARS-CoV-2 population (lower panel). (B) Diagram of the Spike ORF, including a potential transframe product. (C) Sequence alignment of amino acids 1071−1086 of Spike, and alignment of the corresponding nucleotide sequences show emergence of a new TRS-B sequence. (D) The sequence context of the novel Spike.iORF sgmRNA, showing TRS-B (blue highlight), extended homology to the 5′UTR (green highlight) during nascent (–) strand RNA synthesis (black), and downstream tandem start codons and Kozak contexts (yellow highlight). (E) Phylogenetic reconstruction of SARS-CoV-2 evolution in humans, with independent emergences of Spike.iORF TRS sequence with ≥50 descendant genomes highlighted in pink (see S1 Table). The schematic and abbreviations of Spike protein domains shown in panel B was adapted from Lan and colleagues [16]. TRS, transcription regulatory sequence. (PDF) [file pbio.3002982.s003.pdf]

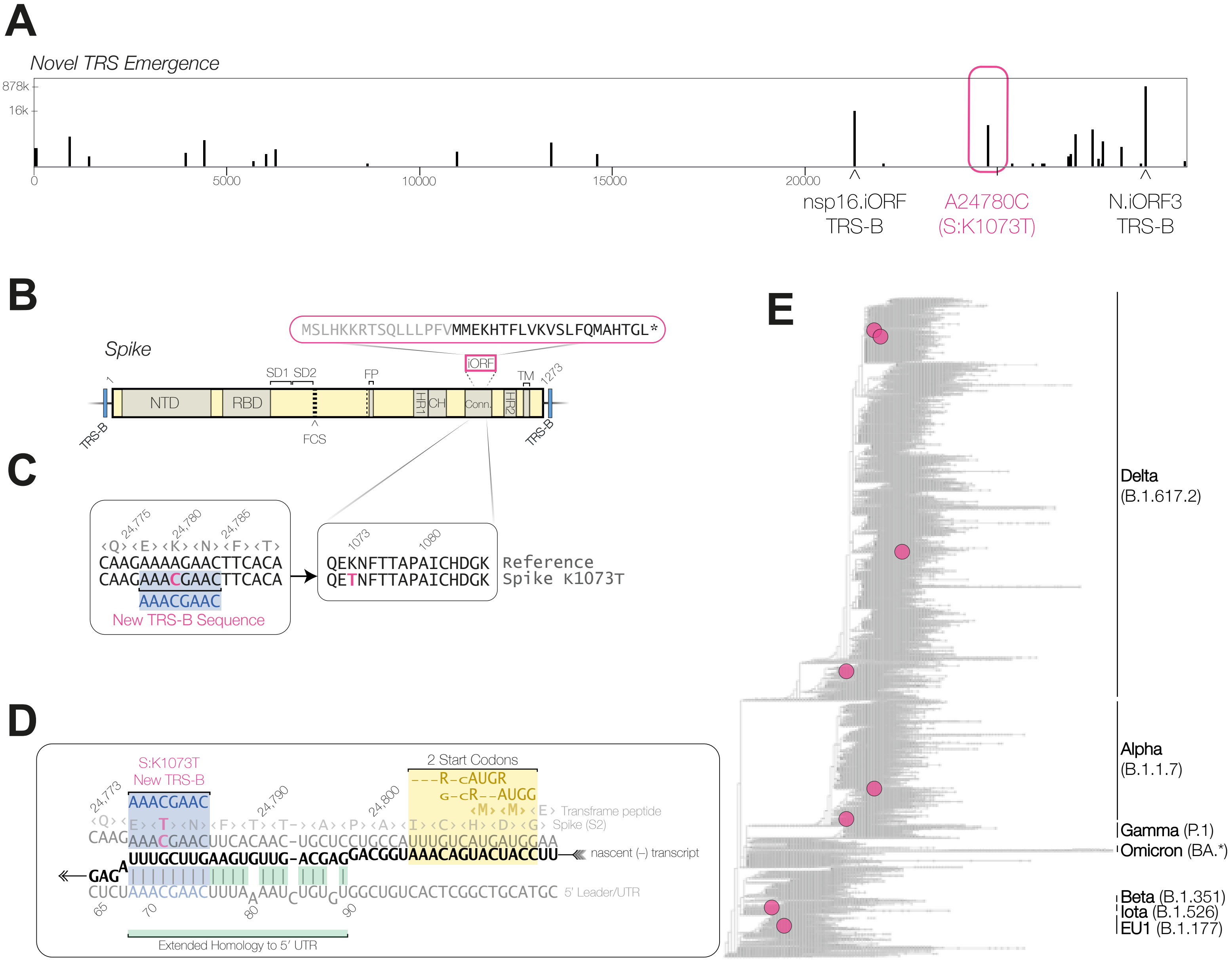

Fig. S3 Convergent evolution of a TRS-B site within the coding region of Spike protein, overlapping the connector domain of Spike/S2. (**A**) Schematic of the SARS-CoV-2 genome (upper panel) and frequency of emergence of the TRS-B sequence (AAACGAAC) in the global SARS-CoV-2 population (lower panel). (**B**) Diagram of the Spike ORF, including a potential transframe product. (**C**) Sequence alignment of amino acids 1071-1086 of Spike, and alignment of the corresponding nucleotide sequences show emergence of a new TRS-B sequence. (**D**) The sequence context of the novel Spike.iORF sgmRNA, showing TRS-B (blue highlight), extended homology to the 5′UTR (green highlight) during nascent (–) strand RNA synthesis (black), and downstream tandem start codons and Kozak contexts (yellow highlight). (**E**) Phylogenetic reconstruction of SARS-CoV-2 evolution in humans, with independent emergences of Spike.iORF TRS sequence with ≥50 descendant genomes highlighted in pink (See **Table S1**). The schematic and abbreviations of Spike protein domains shown in panel b was adapted from Lan et al.(16)
