# Supplementary material for: Emergence of SARS-CoV-2 subgenomic RNAs that enhance viral fitness and immune evasion
Source: PLoS Biol. 2025 Jan 21;23(1):e3002982. doi: 10.1371/journal.pbio.3002982 (PMC11774490; doi:10.1371/journal.pbio.3002982)
Supplement: S4 Fig — (A) Schematic of the SARS-CoV-2 genome (upper panel) and frequency of emergence of the minTRS-B sequence (ACGAAC, middle panel) and full-length TRS-B sequence (AAACGAAC, lower panel) in the global SARS-CoV-2 population. (B) Diagram of the ORF3a, including a potential transframe product, showing two loci of new minTRS-B emergence (i) and (ii), upstream of (iii) the canonical E minTRS-B. (C) Sequence alignment of amino acids 238−275 of ORF3a, and alignment of the corresponding nucleotide sequences show two loci of new minTRS-B emergence. (D) The sequence context of the novel (i) ORF3a.iORF1 and (ii) ORF3a.iORF2 sgmRNA relative to (iii) existing minTRS-B that drives canonical E sgmRNA expression, showing minTRS-B sites (blue highlights), extended homologies to the 5′UTR (green highlights) during nascent (–) strand RNA synthesis (black), and downstream start codons and Kozak contexts (yellow highlights). (E) Phylogenetic reconstruction of SARS-CoV-2 evolution in humans, with independent emergences of ORF3a.iORF minTRS-B sites with ≥50 descendant genomes highlighted in orange, with overlaid (i) or (ii) annotation in white denoting ORF3a.iORF1 minTRS-B and ORF3a.iORF2 minTRS-B emergence, respectively. (F) Percentage of sequenced Delta variant genomes in Australia that contain (ii) ORF3a.iORF2 minTRS-B mutation (See S1 Table). The plot in panel F was generated using CoV-Spectrum [44]. TRS, transcription regulatory sequence. (PDF) [file pbio.3002982.s004.pdf]

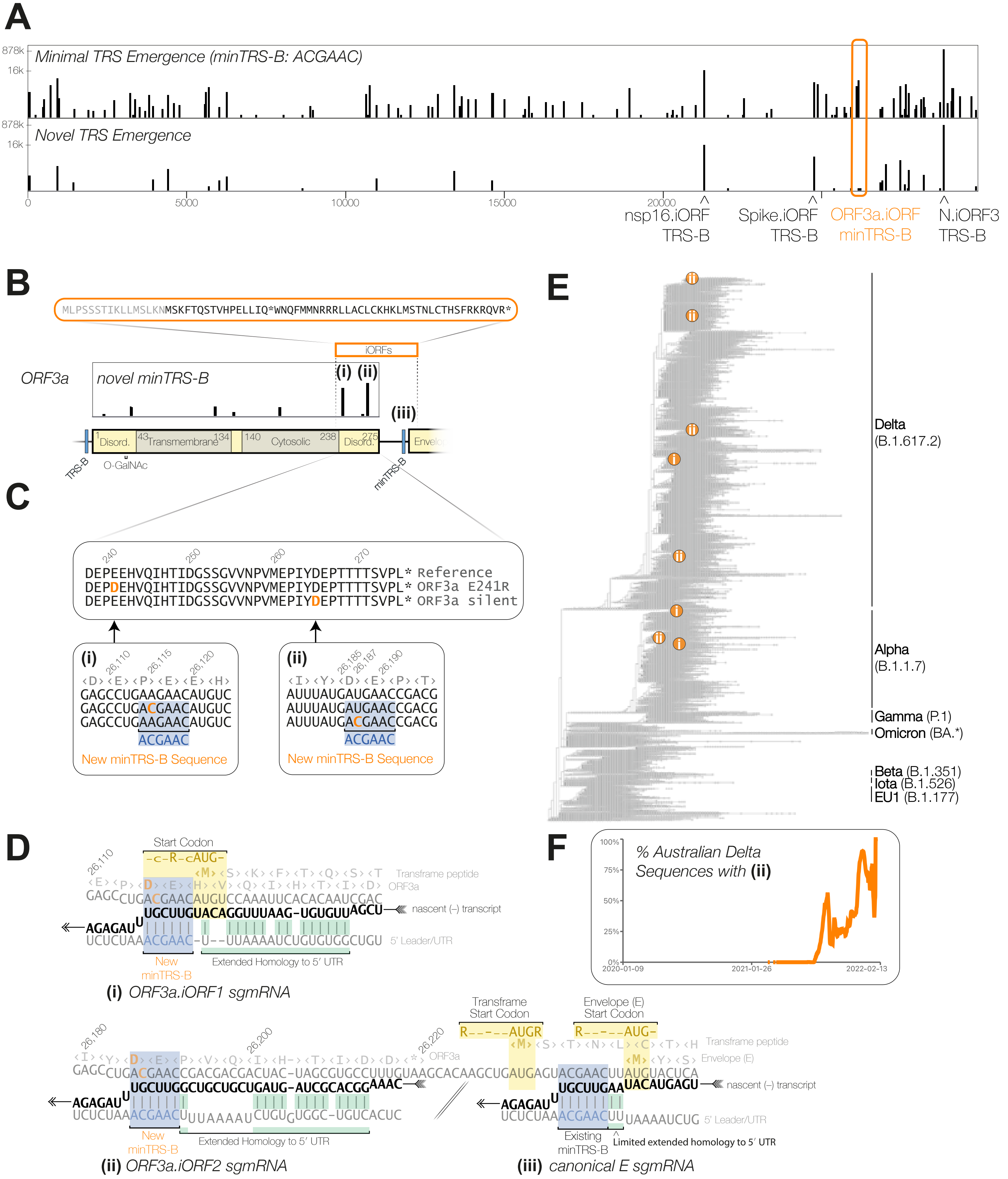

Fig. S4. Evolution of minimal TRS-B (minTRS-B) sites upstream of the canonical Envelope minTRS-B. (**A**), Schematic of the SARS-CoV-2 genome (upper panel) and frequency of emergence of the minTRS-B sequence (ACGAAC, middle panel) and full-length TRS-B sequence (AAACGAAC, lower panel) in the global SARS-CoV-2 population. (**B**), Diagram of the ORF3a, including a potential transframe product, showing two loci of new minTRS-B emergence (i) and (ii), upstream of (iii) the canonical E minTRS-B. (**C**), Sequence alignment of amino acids 238-275 of ORF3a, and alignment of the corresponding nucleotide sequences show two loci of new minTRS-B emergence. (**D**), The sequence context of the novel (i) ORF3a.iORF1 and (ii) ORF3a.iORF2 sgmRNA relative to (iii) existing minTRS-B that drives canonical E sgmRNA expression, showing minTRS-B sites (blue highlights), extended homologies to the 5′UTR (green highlights) during nascent (–) strand RNA synthesis (black), and downstream start codons and Kozak contexts (yellow highlights). (**E**), Phylogenetic reconstruction of SARS-CoV-2 evolution in humans, with independent emergences of ORF3a.iORF minTRS-B sites with ≥50 descendant genomes highlighted in orange, with overlaid (i) or (ii) annotation in white denoting ORF3a.iORF1 minTRS-B and ORF3a.iORF2 minTRS-B emergence, respectively. (**F**), Percentage of sequenced Delta variant genomes in Australia that contain (ii) ORF3a.iORF2 minTRS-B mutation (See **Table S1**). The plot in panel f was generated using CoV-Spectrum (44).
