# Supplementary material for: Emergence of SARS-CoV-2 subgenomic RNAs that enhance viral fitness and immune evasion
Source: PLoS Biol. 2025 Jan 21;23(1):e3002982. doi: 10.1371/journal.pbio.3002982 (PMC11774490; doi:10.1371/journal.pbio.3002982)
Supplement: S5 Fig — (A) Schematic representation of qRT-PCR primer probe sets for N, N.iORF3 and E sgmRNAs and (B) standard curves using synthetic cDNA oligonucleotide templates. Insets show linear regression of Ct plotted against log10-transformed cDNA copy number. (C) RT-qPCR of VeroE6 cells infected with B, B.1.1, Alpha or mock cells, or water controls, validating RT-qPCR specificity. Bars represent the mean and standard deviation of three biological replicates. RT-qPCR analysis of N.iORF3 sgmRNA copy number, expressed as a ratio of N copy number in (D) clinical swabs, and (E) infected VeroE6 cells in culture at 7 and 24 h post-infection. For infected VeroE6 cells, data are means and standard deviations of at least three biological replicates. For clinical swabs, data are means and standard deviations of 4 (EU1/Alpha) or 12 (Delta/Omicron) swab samples per lineage. Data underlying this figure can be found in: https://doi.org/10.25418/crick.27952842. (PDF) [file pbio.3002982.s005.pdf]

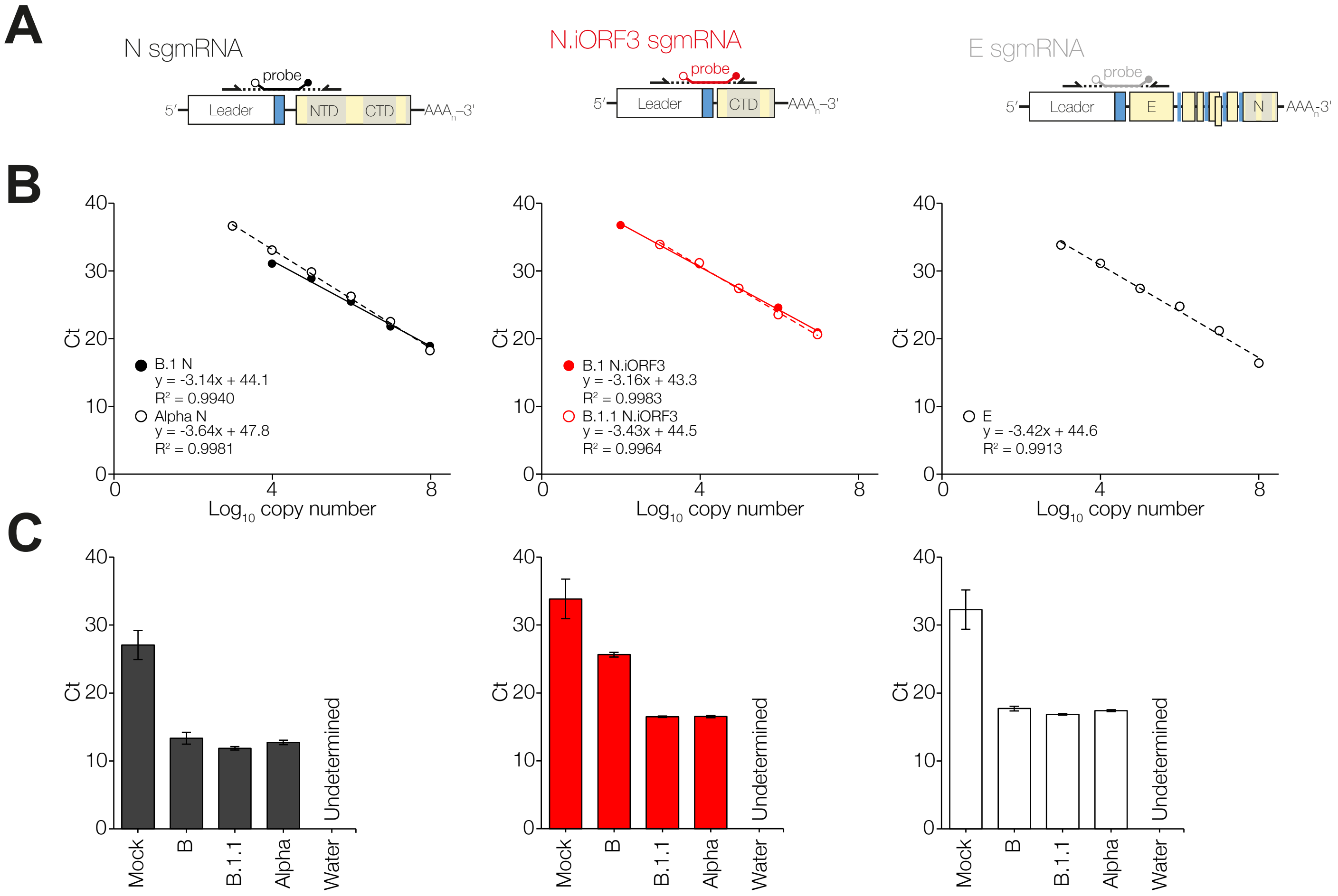

Fig. S5. Validation of sgmRNA RT-qPCR and determination of sgmRNA copy number. (**A**) Schematic representation of qRT-PCR primer probe sets for N, N.iORF3 and E sgmRNAs, and (**B**) standard curves using synthetic cDNA oligonucleotide templates. Insets show linear regression of Ct plotted against log10-transformed cDNA copy number. (**C**) RT-qPCR of VeroE6 cells infected with B, B.1.1, Alpha or mock cells, or water controls, validating RT-qPCR specificity. Bars represent the mean and standard deviation of three biological replicates. RT-qPCR analysis of N.iORF3 sgmRNA copy number, expressed as a ratio of N copy number in (**D**) clinical swabs, and (**E**) infected VeroE6 cells in culture at 7 and 24 hours post-infection. For infected VeroE6 cells, data are means and standard deviations of at least three biological replicates. For clinical swabs, data are means and standard deviations of four (EU1/Alpha) or twelve (Delta/Omicron) swab samples per lineage. Data underlying this figure can be found in: https://doi.org/10.25418/crick.27952842.
