# Supplementary material for: Emergence of SARS-CoV-2 subgenomic RNAs that enhance viral fitness and immune evasion
Source: PLoS Biol. 2025 Jan 21;23(1):e3002982. doi: 10.1371/journal.pbio.3002982 (PMC11774490; doi:10.1371/journal.pbio.3002982)
Supplement: S6 Fig — (A, B) N.iORF3 copy number expressed as a percentage of Nucleocapsid copy number. (C–H) Expression of Envelope (B), Nucleocapsid (C) or N.iORF3 (D) sgmRNA, normalised to genomic RNA (ORF1ab) and expressed as fold change compared to the control virus condition: for swab samples (A, C, E, G), EU1, and for infections in VeroE6 cells (B, D, F, H), B lineage. Alpha-ins represents an additional Alpoha isolate with an insertion upstream of the Nucleocapsid codiing region, described in S8 Fig. Data underlying this figure can be found in: https://doi.org/10.25418/crick.27952842. (PDF) [file pbio.3002982.s006.pdf]

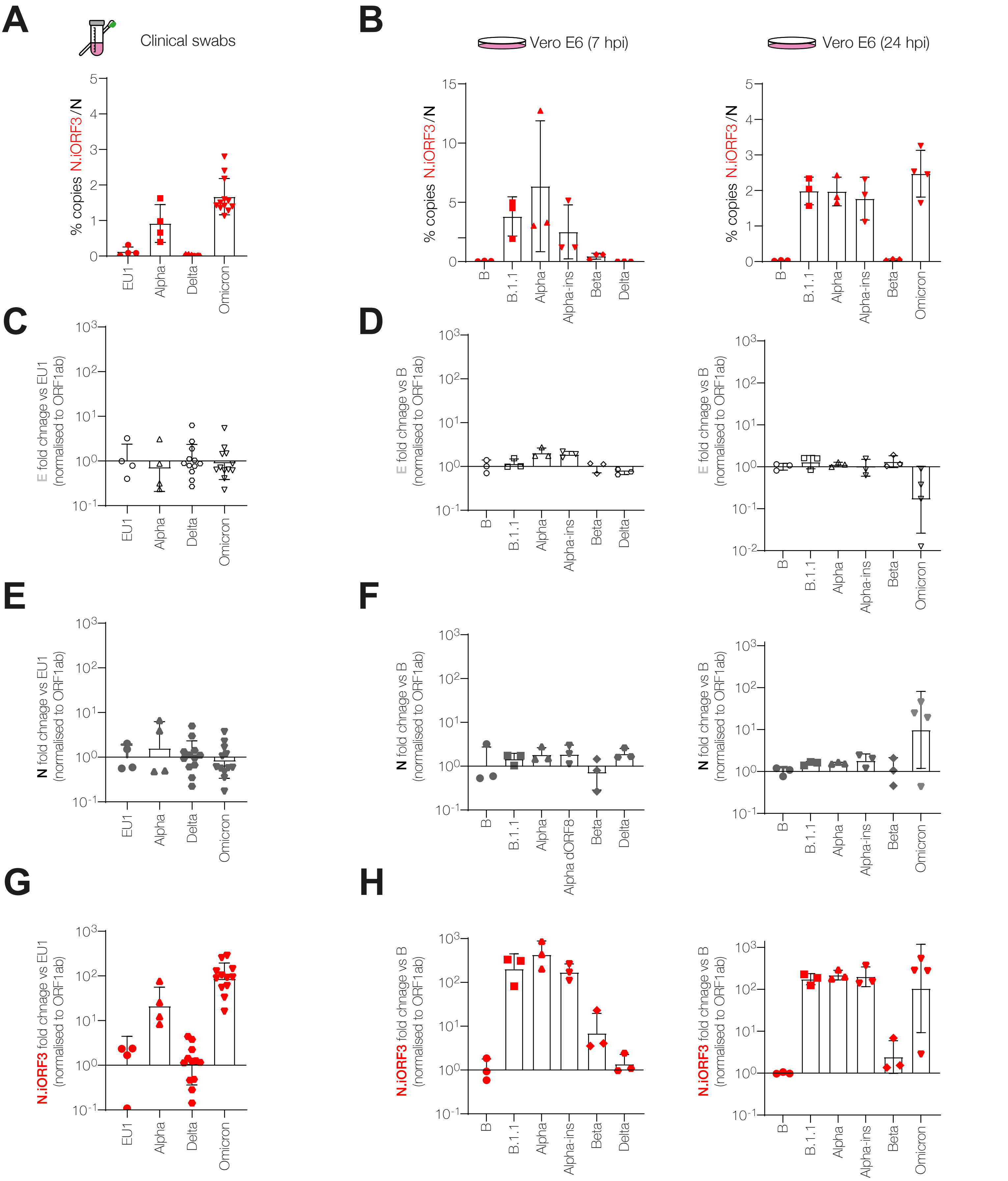

Fig. S6. Extended RT-qPCR analysis of sgmRNA expression in SARS-CoV-2 infected clinical swabs and cell culture. (**A,B**) N.iORF3 copy number expressed as a percentage of Nucleocapsid copy number. (**C-H**) Expression of Envelope (**B**), Nucleocapsid (**C**) or N.iORF3 (**D**) sgmRNA, normalised to genomic RNA (ORF1ab) and expressed as fold change compared to the control virus condition: for swab samples (**A,C,E,G**), EU1, and for infections in VeroE6 cells (**B,D,F,H**), B lineage. Alpha-ins represents an additional Alpha isolate with an insertion upstream of the Nucleocapsid codiing region. Data underlying this figure can be found in: https://doi.org/10.25418/crick.27952842.
