# Supplementary material for: Emergence of SARS-CoV-2 subgenomic RNAs that enhance viral fitness and immune evasion
Source: PLoS Biol. 2025 Jan 21;23(1):e3002982. doi: 10.1371/journal.pbio.3002982 (PMC11774490; doi:10.1371/journal.pbio.3002982)
Supplement: S7 Fig — (A) Schematic representation of qRT-PCR primer probe sets for N and ORF9b sgmRNAs and (B) standard curve using synthetic cDNA oligonucleotide templates for ORF9b sgmRNA. Standard curve for N sgmRNA is shown in Extended Data Fig 1B. (C) Amplification of N (black) or ORF9b (purple) sgmRNA in Vero E6 cells infected with B, B.1.1 or Alpha, in technical duplicate, representative of three biological replicates. (D) Gel electrophoresis of RT-PCR products from ORF9b RT-qPCR reactions. Data underlying this figure can be found in: https://doi.org/10.25418/crick.27952842 and https://doi.org/10.25418/crick.27953013. (PDF) [file pbio.3002982.s007.pdf]

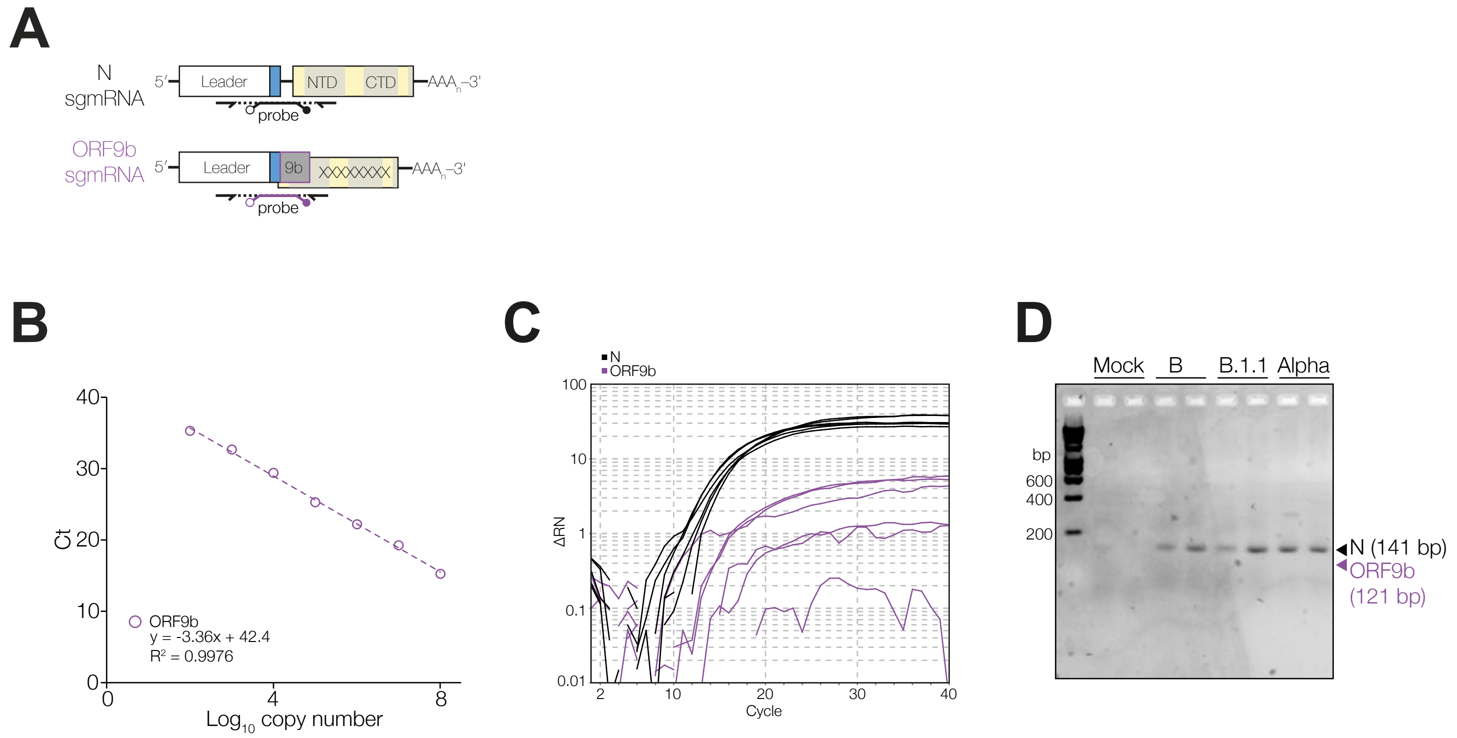

Fig. S7. ORF9b-specific sgmRNA RT-qPCR. (**A**) Schematic representation of qRT-PCR primer probe sets for N and ORF9b sgmRNAs, and (**B**), standard curve using synthetic cDNA oligonucleotide templates for ORF9b sgmRNA. Standard curve for N sgmRNA is shown in Extended Data Fig. 1b. (**C**), Amplification of N (black) or ORF9b (purple) sgmRNA in Vero E6 cells infected with B, B.1.1 or Alpha, in technical duplicate, representative of three biological replicates. (**D**), Gel electrophoresis of RT-PCR products from ORF9b RT-qPCR reactions.Data underlying this figure can be found in: https://doi.org/10.25418/crick.27952842 and https://doi.org/10.25418/crick.27953013.
