# Supplementary material for: Emergence of SARS-CoV-2 subgenomic RNAs that enhance viral fitness and immune evasion
Source: PLoS Biol. 2025 Jan 21;23(1):e3002982. doi: 10.1371/journal.pbio.3002982 (PMC11774490; doi:10.1371/journal.pbio.3002982)
Supplement: S8 Fig — (A, B) Western blot analysis of Mock, B-lineage or Alpha-infected VeroE6 cells, at 24 h post-infection without treatment (A) or at 16 h post-infection with or without 8 h MG-132 treatment (B). (C) Western blot analysis of Mock VeroE6 cell or cells infected with reverse-genetics-derived viruses as indicted (see Figs 5 and S9). MW, molecular weight marker. Data underlying this figure can be found in: https://doi.org/10.25418/crick.27953013. (PDF) [file pbio.3002982.s008.pdf]

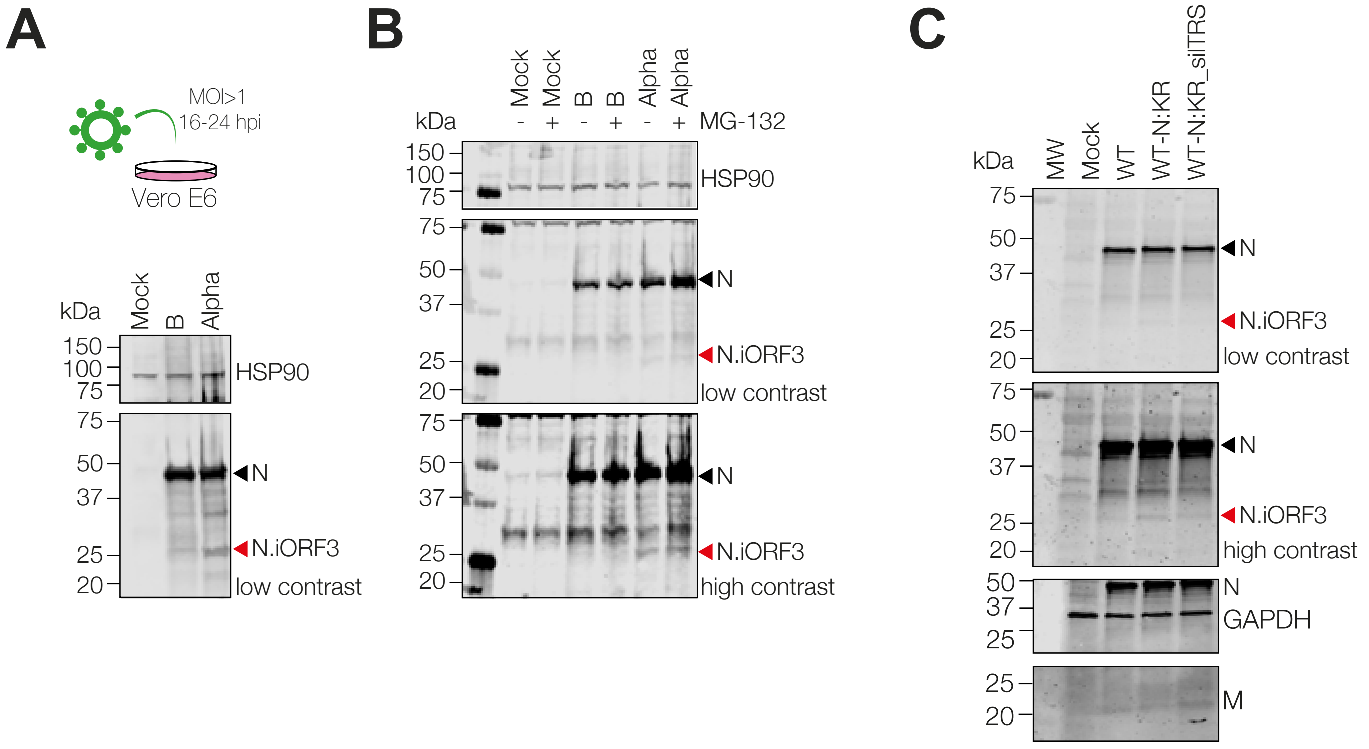

Fig. S8. Expression of N.iORF3 protein in infection. (**A-B**) Western blot analysis of Mock, B-lineage or Alpha-infected VeroE6 cells, at 24 hours post infection without treatment (**A**) or at 16 hours post infection with or without 8 hours MG-132 treatment (**B**). (**C**) Western blot analysis of Mock VeroE6 cell or cells infected with reverse-genetics derived viruses as indicted (see Fig. 5 and S9). MW, molecular weight marker. Data underlying this figure can be found in: https://doi.org/10.25418/crick.27953013.
