# Supplementary material for: Emergence of SARS-CoV-2 subgenomic RNAs that enhance viral fitness and immune evasion
Source: PLoS Biol. 2025 Jan 21;23(1):e3002982. doi: 10.1371/journal.pbio.3002982 (PMC11774490; doi:10.1371/journal.pbio.3002982)
Supplement: S9 Fig — (A) Summary of reverse genetics mutants used in the Alpha backbone, showing nucleotide mutations and corresponding amino acid changes (left panel) and schematic of the experimental design (right panel). (B–D) reverse transcription qPCR (RT-qPCR) analysis of the indicated sgmRNAs, normalised to ORF1ab and expressed as fold change relative to expression in Alpha-WT. Log10-transfrmed values were compared by one-way ANOVA with Tukey’s multiple comparisons test. Data underlying this figure can be found in: https://doi.org/10.25418/crick.27952842. (PDF) [file pbio.3002982.s009.pdf]

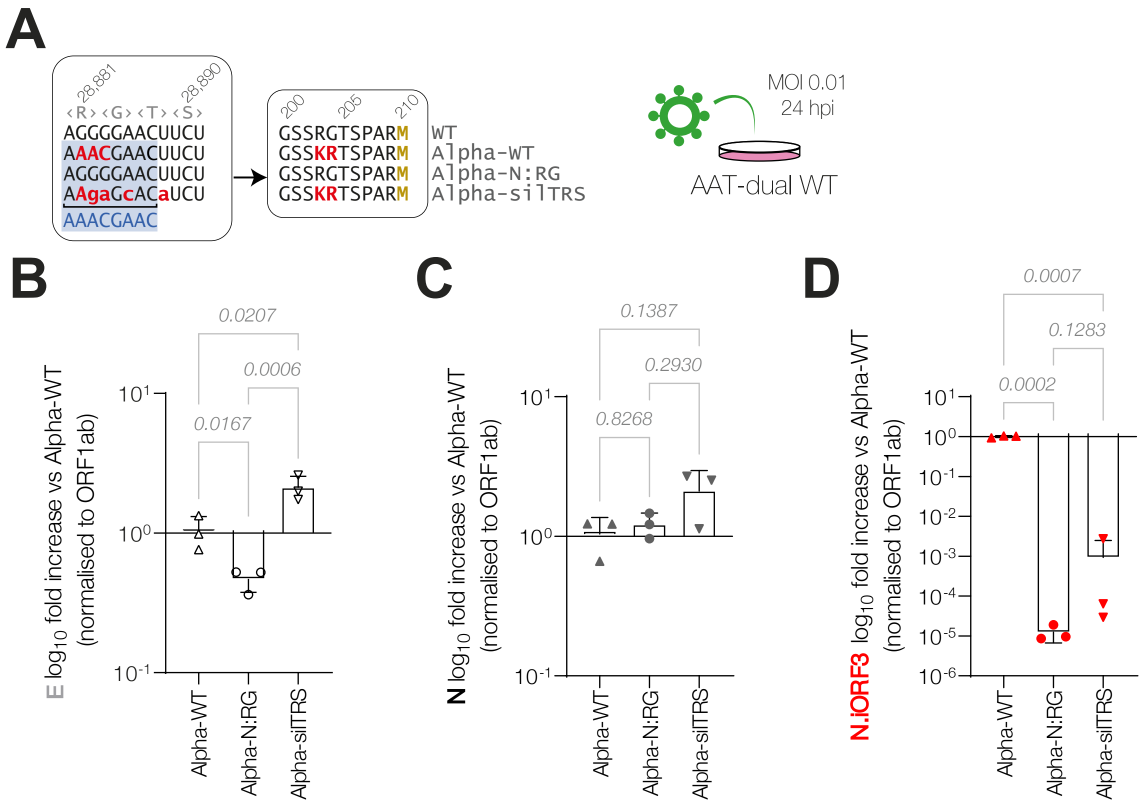

Fig. S9. sgmRNA expression in reverse-genetics-derived viruses. (**A**) Summary of reverse genetics mutants used in the Alpha backbone, showing nucleotide mutations and corresponding amino acid changes (left panel) and schematic of the experimental design (right panel). (**B-D**) RT-qPCCR analysis of the idicated sgmRNAs, normalised to ORF1ab and expressed as fold change relative to expression in Alpha-WT. Log10-transfrmed values were compared by one-way ANOVA with Tukey's multiple comparisons test. Data underlying this figure can be found in: https://doi.org/10.25418/crick.27952842.
