# Supplementary material for: Emergence of SARS-CoV-2 subgenomic RNAs that enhance viral fitness and immune evasion
Source: PLoS Biol. 2025 Jan 21;23(1):e3002982. doi: 10.1371/journal.pbio.3002982 (PMC11774490; doi:10.1371/journal.pbio.3002982)
Supplement: S10 Fig — (A) Summary of reverse genetics mutants used in the WT (Wuhan-Hu-1 S:D614G) backbone, showing nucleotide mutations and corresponding amino acid changes (left panel) and schematic of the experimental design (right panel). (B) Growth of mutant viruses individually, measured by reverse transcription qPCR (RT-qPCR) against ORF1ab, normalised to actin and (C) corresponding area under the curve (AUC) values. Data are means and standard deviations of three biological replicates, compared to WT-N:KR by one-way ANOVA (C). (D, E) Head-to-head competition assay comparing fitness Alpha-WT and Alpha-N:RG viruses (D), or Alpha-N:RG and Alpha-silTRS viruses (E), measured by Illumina sequencing of amplicons spanning the N.iORF3 TRS-B region and expressed as percentage of WT-N:KR reads. Total ORF1ab expression, normalised to actin, is shown on the right y-axes for reference. Data underlying this figure can be found in: https://doi.org/10.25418/crick.27952842. (PDF) [file pbio.3002982.s010.pdf]

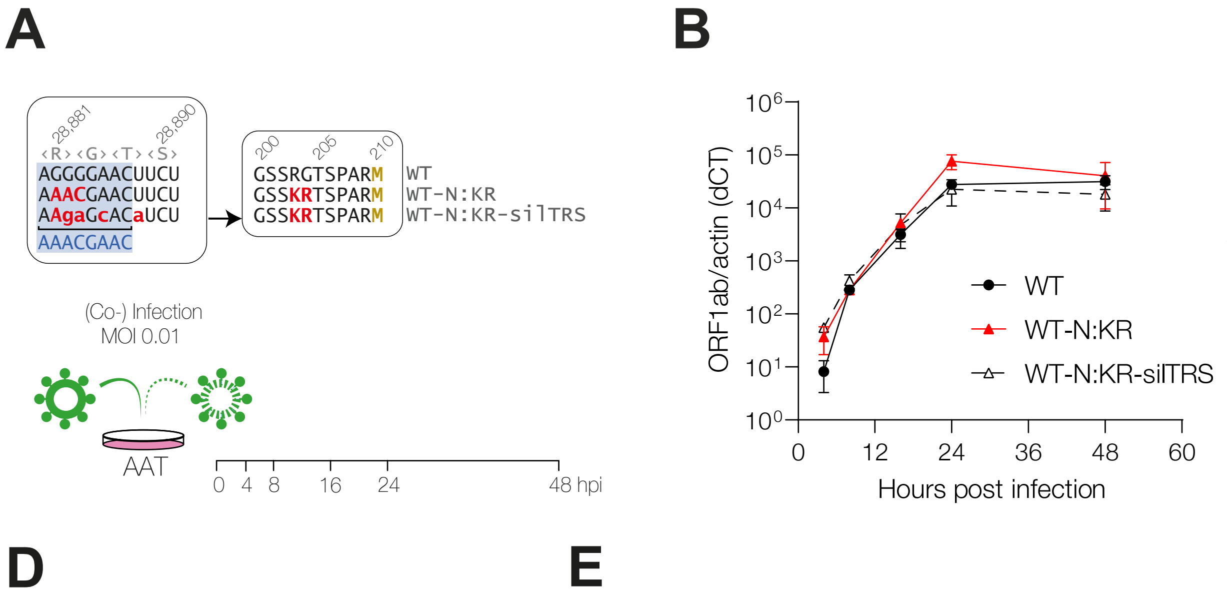


**Fig. S10. Growth of WT- and Alpha-backbone virus mutants.** (**A**) Summary of reverse genetics mutants used in the WT (Wuhan-Hu-1 S:D614G) backbone, showing nucleotide mutations and corresponding amino acid changes (left panel) and schematic of the experimental design (right panel). (**B**) Growth of mutant viruses individually, measured by RT-qPCR against ORF1ab, normalised to actin and (**C**) corresponding area under the curve (AUC) values. Data are means and standard deviations of three biological replicates, compared to WT-N:KR by one-way ANOVA (**C**). (**D-E**) Head-to-head competition assay comparing fitness Alpha-WT and Alpha-N:RG viruses (**D**), or Alpha-N:RG and Alpha-silTRS viruses (**E**), measured by Illumina sequencing of amplicons spanning the N.iORF3 TRS-B region and expressed as percentage of WT-N:KR reads. Total ORF1ab expression, normalised to actin, is shown on the right y axes for reference. Data underlying this figure can be found in: https://doi.org/10.25418/crick.27952842.
