# Supplementary material for: Emergence of SARS-CoV-2 subgenomic RNAs that enhance viral fitness and immune evasion
Source: PLoS Biol. 2025 Jan 21;23(1):e3002982. doi: 10.1371/journal.pbio.3002982 (PMC11774490; doi:10.1371/journal.pbio.3002982)
Supplement: S11 Fig — (A–C) Growth of Alpha-WT (A), Alpha-N:RG (B) or Alpha-silTRS (C) in A549-dual ACE2-TMPRSS2 cells (WT), MDA5 knockout (MDA5 KO) or RIG-I KO cells, measured by reverse transcription qPCR (RT-qPCR) against ORF1ab, normalised to 18S rRNA and (D) corresponding area under the curve (AUC) values. Data are means and standard deviations of three biological replicates, compared by two-way ANOVA with Tukey’s multiple comparisons. P-values are shown. One-way ANOVA comparisons of individual time points for each virus are presented in S3 Table. NB: Growth curve data and AUC values for Alpha-WT in WT cells, Alpha-WT in RIG-I KO cells, Alpha-silTRS in WT cells and Alpha-silTRS in KO cells are the same as presented in Fig 6B and C. (PDF) [file pbio.3002982.s011.pdf]

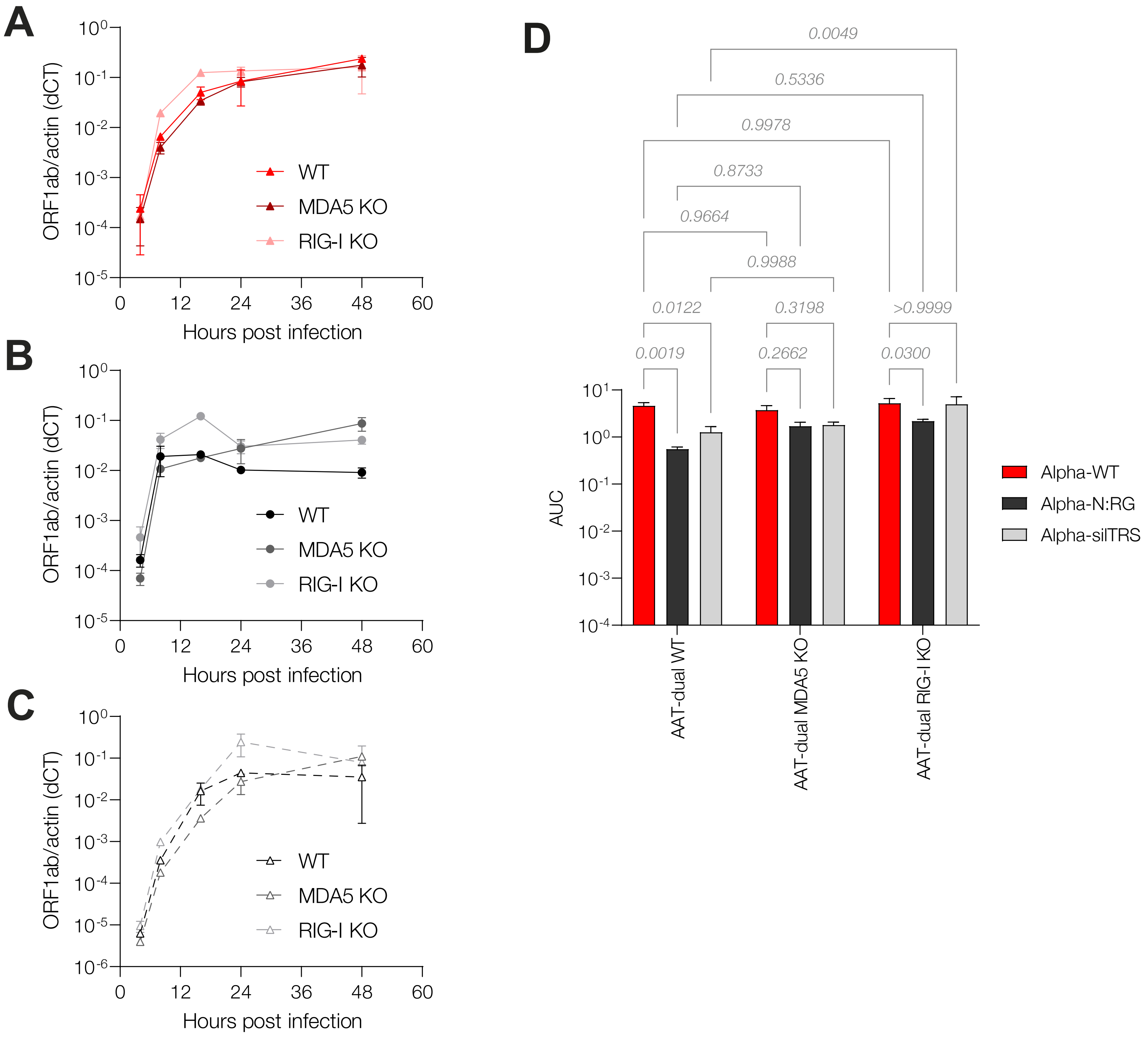


**Fig. S11. Growth of WT- and Alpha-backbone virus mutants in MDA5 KO and RIG-I KO cells.** (**A-C**) Growth of Alpha-WT (A), Alpha-N:RG (B) or Alpha-silTRS (C) in A549-dual ACE2-TMPRSS2 cells (WT), MDA5 knockout (MDA5 KO) or RIG-I KO cells, measured by RT-qPCR against ORF1ab, normalised to 18S rRNA and (**D**) corresponding area under the curve (AUC) values. Data are means and standard deviations of three biological replicates, compared by two-way ANOVA with Tukey's multiple comparisons. P values are shown. One-way ANOVA comparisons of individual time points for each virus are presented in Table S3. NB: Growth curve data and AUC values for Alpha-WT in WT cells, Alpha-WT in RIG-I KO cells, Alpha-silTRS in WT cells and Alpha-silTRS in KO cells are the same as presented in Figure 6B and 6C. Data underlying this figure can be found in: https://doi.org/10.25418/crick.27952842.
