# Supplementary material for: Emergence of SARS-CoV-2 subgenomic RNAs that enhance viral fitness and immune evasion
Source: PLoS Biol. 2025 Jan 21;23(1):e3002982. doi: 10.1371/journal.pbio.3002982 (PMC11774490; doi:10.1371/journal.pbio.3002982)
Supplement: S2 Table — Data were log-transformed, six biological replicates across two independent experiments. P-values less than 0.05 are highlighted in bold. hpi, hours post-infection. (DOCX) [file pbio.3002982.s013.docx]

**Table S2. One-way ANOVA analysis with Tukey’s multiple comparisons test of individual timepoints for data shown in Fig. 5B.** Data were log-transformed, six biological replicates across two independent experiments. P values less than 0.05 are highlighted in bold. hpi, hours post infection.

| Comparison | 4 hpi | 8 hpi | 16 hpi | 24 hpi | 48 hpi |
| --- | --- | --- | --- | --- | --- |
| Alpha-WT vs. Alpha-N:RG | **0.0001** | **0.0003** | 0.0872 | **0.0083** | **<0.0001** |
| Alpha-WT vs. Alpha-silTRS | **<0.0001** | **0.0014** | **0.0500** | 0.0577 | 0.9014 |
| Alpha-N:RG vs. Alpha-silTRS | 0.7778 | **<0.0001** | **0.0005** | 0.5929 | **<0.0001** |
