# Supplementary material for: Emergence of SARS-CoV-2 subgenomic RNAs that enhance viral fitness and immune evasion
Source: PLoS Biol. 2025 Jan 21;23(1):e3002982. doi: 10.1371/journal.pbio.3002982 (PMC11774490; doi:10.1371/journal.pbio.3002982)
Supplement: S3 Table — Comparisons were carried out for each virus, comparing genome expression at each time point in KO cell lines compared to WT cells. Data were log-transformed, three biological replicates. P-values less than 0.05 are highlighted in bold. hpi, hours post-infection. (DOCX) [file pbio.3002982.s014.docx]

**Table S3. One-way ANOVA analysis with Tukey’s multiple comparisons test of individual timepoints for data shown in Fig 6B and S10.** Comparisons were carried out for each virus, comparing genome expression at each time point in KO cell lines compared to WT cells. Data were log-transformed, three biological replicates. P values less than 0.05 are highlighted in bold. hpi, hours post infection.

| Comparison | 4 hpi | 8 hpi | 16 hpi | 24 hpi | 48 hpi |
| --- | --- | --- | --- | --- | --- |
| Alpha-WT: WT vs MDA5 KO | 0.7923 | **0.0271** | 0.1523 | 0.9089 | 0.7534 |
| Alpha-WT: WT vs RIG-I KO | 0.9942 | **0.0007** | **0.0036** | 0.247 | 0.4181 |
| Alpha-N:RG: WT vs MDA5 KO | 0.0729 | 0.3082 | 0.2495 | **0.0266** | 0.2874 |
| Alpha-N:RG: WT vs RIG-I KO | **0.0471** | 0.0614 | **<0.0001** | **0.0143** | 0.8624 |
| Alpha-silTRS: WT vs MDA5 KO | **0.0365** | **0.0013** | **0.0155** | 0.2858 | 0.2858 |
| Alpha-silTRS: WT vs RIG-I KO | 0.0734 | **0.0002** | 0.7299 | **0.0105** | **0.0105** |
