# Supplementary material for: Emergence of SARS-CoV-2 subgenomic RNAs that enhance viral fitness and immune evasion
Source: PLoS Biol. 2025 Jan 21;23(1):e3002982. doi: 10.1371/journal.pbio.3002982 (PMC11774490; doi:10.1371/journal.pbio.3002982)
Supplement: S4 Table — (DOCX) [file pbio.3002982.s015.docx]

**Table S4. Primer sequences.**

| Experiment | Primer | Sequence |
| --- | --- | --- |
| RT-PCR | 5′UTR-FWD | 5′-CAGGTAACAAACCAACCAACTTTCG-3′ |
| RT-PCR | ORF9-REV | 5′-TCCTTGTCTGATTAGTTCCTGGTCC-3′ |
| RT-PCR | ORF2-REV | 5′-AATAAGTAGGGACTGGGTCTTCG-3′ |
| RT-qPCR | 5′UTR-FWD | 5′-GTAACAAACCAACCAACTTTCG-3′ |
| RT-qPCR | ORF9-REV | 5′-GGTTACTGCCAGTTGAATCTG-3′ |
| RT-qPCR | N.iORF3-REV | 5′-TGGTTCAATCTGTCAAGCAG-3′ |
| RT-qPCR | ORF4-REV | 5′-GGTTACTGCCAGTTGAATCTG-3′ |
| RT-qPCR | ORF9-probe | 5′-FAM-TTGTAGATCTGTTCTCTAAACGAACAAACTAAATGTCT-BHQ-3′ |
| RT-qPCR | ORF9b-probe | 5′-FAM-TTGTAGATCTGTTCTCTAAATGGACCCCAA-BHQ-3′ |
| RT-qPCR | N.iORF3-probe | 5′-FAM-TAGATCTGTTCTCTAAACGAACTTCTCCTGCTAGAAT-BHQ-3′ |
| RT-qPCR | ORF4-probe | 5′-FAM-AGATCTGTTCTCTAAACGAACTTATGTACTCATTCGTT-BHQ-3′ |
| RT-qPCR | IFNb-FWD | 5′-ACATCCCTGAGGAGATTAAGCA-3′ |
| RT-qPCR | IFNb-REV | 5′-GCCAGGAGGTTCTCAACAATAG-3′ |
| RT-qPCR | IFIT1-FWD | 5′-CCTGAAAGGCCAGAATGAGG-3′ |
| RT-qPCR | IFIT1-REV | 5′-TCCACCTTGTCCAGGTAAGT-3′ |
| RT-qPCR | GAPDH-FWD | 5′-ACCCAGAAGACTGTGGATGG-3′ |
| RT-qPCR | GAPDH-REV | 5′-TTCTAGACGGCAGGTCAGGT-3′ |
