# Supplementary material for: Emergence of SARS-CoV-2 subgenomic RNAs that enhance viral fitness and immune evasion
Source: PLoS Biol. 2025 Jan 21;23(1):e3002982. doi: 10.1371/journal.pbio.3002982 (PMC11774490; doi:10.1371/journal.pbio.3002982)
Supplement: S1 Table — Identified nodes on a phylogenetic reconstruction of SARS-CoV-2 evolution in humans representing clusters of novel TRS and sgmRNA emergence (see Materials and methods). For each emergence event, the parental lineage, approximate date (“Approx. Date”) and location (“Location Focus”) of circulation are annotated, as well as number of identified descendant genomes within each cluster (“N=”), whether it appears to be a true independent emergence event (“Indep?”, see Materials and methods) and whether it has further extended homology to the 5′UTR (“+Homol?”). Each cluster is further annotated with potential interrelationships (“Links”) and additional notes, including context to its spread based on analysis using CoV-Spectrum [29]. (PDF) [file pbio.3002982.s012.pdf]

N.IORF3 TRS-B — *nodes with ≥50 descendant genomes are listed*

Taxonium mutations: G28881A & G28882A & G28883C
CoV-Spectrum query: LINEAGE* & G28881A & G28882A & G28883C — *≥2 independent emegence events*
Amino acid mutations: R203K, G204R

Variant / Lineage	Approx. Date	Location Focus	N=	Indep?	+Homol?	Links	Notes
**B.1.1***	Jan 2020 - Present	Unknown	2855835	Yes	*see below*	may have arisen convergently	*Entire Lineage; (5% advantage Mar-Jun 2020)*
**Iota (B.1.526)**	Feb-July 2021	USA (FL, PA)	140	Yes	No		

N.IORF3 + Extended Homology TRS-B — *nodes with ≥50 descendant genomes are listed*

Taxonium mutations: 28877T & 28878C & G28881A & G28882A & G28883C
CoV-Spectrum query: LINEAGE* & 28877T & 28878C & G28881A & G28882A & G28883C — *≥13 independent emegence events*
Amino acid mutations: S202S, R203K, G204R

Variant / Lineage	Approx. Date	Location Focus	N=	Indep?	+Homol?	Links	Notes
**Gamma (P.1)**	Early 2021	Brazil	89722	Yes	Yes		*Entire Lineage*
**B.1.560**	Early 2021	India	237	Yes	Yes		
**B.1.1.416**	Dec-Jan 2020-1	USA	50	Yes	Yes		*5% of B.1.1.416*
**Omicron (BA.1.1)**	Dec-Jan 2021-2	S Korea / Europe	50	Yes	Yes		*BA.1.1 overall: steady rise from 0% to 5% Nov 2021-Feb 2022*
**Lambda (C.37.1)**	Summer 2020	Spain	56	Yes	Yes		*85%+ of Lineage*
**B.1.1.519**	Early Spring 2021	USA	550	Yes	Yes		
**B.1.1.214**							
B.1.1.214 (cluster 1)	Nov-Feb 2020-1	Japan	175	Yes	Yes		
B.1.1.214 (cluster 2)	Nov-Feb 2020-1	Japan	50		Yes	likely linked w/cluster 1	
**Alpha (B.1.1.7)**							*Alpha overall: steady rise from 0% to 2.5% Dec 2020 to Jul 2021 (to 8% in USA)*
B.1.1.7 (cluster 1)	Spring 2021	Japan	50	Yes	Yes		
B.1.1.7 (cluster 2)	Spring 2021	USA, Spain	7172	Yes	Yes		
B.1.1.7 (cluster 3)	Feb-Mar 2021	UK	165	Yes	Yes		
B.1.1.7 (cluster 4)	Feb-Mar 2021	UK	59		Yes	likely linked w/cluster 3	
B.1.1.7 (cluster 5)	Mar-Jul 2021	Denmark, Poland	366	Yes	Yes		
B.1.1.7 (cluster 6)	Apr-Jul 2021	USA	175	Yes	Yes		
B.1.1.7 (cluster 7)	Feb-May 2021	USA, Mexico	62		Yes	likely linked w/cluster 6	
B.1.1.7 (cluster 8)	May-June 2021	Norway	81		Yes	likely linked w/cluster 9	
B.1.1.7 (cluster 9)	Feb-June 2021	Sweden, France, Europe	4177	Yes	Yes		
B.1.1.7 (cluster 10)	Feb-Apr 2021	Sweden	128		Yes	possibly linked w/cluster 9	
B.1.1.7 (cluster 11)	Feb-Apr 2021	Sweden	128		Yes	possibly linked w/cluster 9	
B.1.1.7 (cluster 12)	Mar-May 2021	USA	119		Yes		

nsp16.IORF TRS-B — *nodes with ≥100 descendant genomes are listed*

Taxonium mutations: C21304A & G21305A & C21302T (exended homology)
CoV-Spectrum query: LINEAGE* & C21304A & G21305A — *≥21 independent emegence events*
Amino acid mutations: 2613N (ORF1ab: 7014N) & P2612L (exended homology)

Variant / Lineage	Approx. Date	Location Focus	N=	Indep?	+Homol?	Links	Notes
**B.1.243**	Nov-Jan 2020-1	USA	228	Yes	No		
**EU1 (B.1.177.80)**	Dec-Mar 2020-1	Scandinavia	448	Yes	No		
**Iota (B.1.526)**	Apr-May 2021	USA	109	Yes	No		
**B.1.2**	Dec-Feb 2020-1	USA	974	Yes	ALL		
**B.1.1.44**	Aug-Oct 2020	UK	206	Yes	ALL		
**Alpha (B.1.1.7)**							
B.1.1.7 (cluster 1)	Mar-Jun 2021	Canada	2906	Yes	ALL		*Canada Alpha overall: steady rise from 0% to 30% (13% adv)*
B.1.1.7 (cluster 2)	Apr-Jun 2021	UK	350	Yes	No		
B.1.1.7 (cluster 3)	Mar-Jun 2021	UK, Central Europe	158		No	possibly linked w/cluster 2	*other sequences possibly misplaced with this cluster*
B.1.1.7 (cluster 4a)	Jan-May 2021	UK, Japan, Central Europe	140	Yes	No		
B.1.1.7 (cluster 4b)	Jan-Mar 2021	UK, Norway, Japan	308		Yes	descendant of cluster 4a	
**Delta (B.1.617.2)**							*global Delta overall: steady rise from 0% to 1% of seqs (5% adv)*
AY.69 (cluster 1)	Oct-Dec 2021	South Korea	167	Yes	No		
AY.23 (cluster 2)	Jun-Dec 2021	Indonesia, Malaysia	620	Yes	No		
AY.44 (cluster 3a)	Jul-Nov 2021	USA	113	Yes	No		
AY.44 (cluster 3b)	Jul-Oct 2021	USA	208		Yes	descendant of cluster 3a	
AY.103 (cluster 4)	Jul-Dec 2021	USA	587	Yes	Yes		*plus 10 ascendants w/o frthr. homology; long duration*
AY.4 (cluster 5a)	Aug-Oct 2021	UK	948	Yes	No		
AY.4 (cluster 5b)	Aug-Nov 2021	UK	208		Yes	descendant of cluster 5a	*incl. possible misplaced sequences from AY.39.1, AY.110*
AY.105 (cluster 6)	Aug-Nov 2021	USA	161	Yes	ALL		
AY.112 (cluster 7)	Oct-Nov 2021	Austria, Germany	125	Yes	No		*Partial TRS-B (21304A) in 880 parental sequences*
AY.29 (cluster 8)	Aug-Sep 2021	Japan	118	Yes	No		
AY.4.2 (cluster 9)	Oct-Dec 2021	UK	5708	Yes	No		
AY.3.1 / AY.100 (cluster 10)	Jul-Dec 2021	USA	1608	Yes	ALL		*incl. possible misassigned sequences to AY.100*
AY.3 (cluster 11)	Aug-Dec 2021	USA	237	Yes	No		
AY.4.8 (cluster 12)	Sep-Dec 2021	UK	616	Yes	ALL		
AY.4 (cluster 13)	Aug-Dec 2021	UK	100	Yes	No		*plus 86 additional sequences nearby on tree in AY.4*
AY.4 (cluster 14)	Nov-Dec 2021	UK	104		ALL	possibly linked w/cluster 13	

Spike.IORF TRS-B — *nodes with ≥50 descendant genomes are listed*

Taxonium mutations: A24780C
CoV-Spectrum query: LINEAGE* & A24780C — *≥7 independent emegence events*
Amino acid mutations: K1073T

Variant / Lineage	Approx. Date	Location Focus	N=	Indep?	Links	Notes
**EU1 (B.1.177.12)**	Dec-Feb 2020-1	Denmark	1218	Yes		*rises from 0% to 20% of lineage Nov-Jan 2020-1*
**B.1.369**	Nov-Mar 2020-1	USA	69	Yes		
**Alpha (B.1.1.7)**						
B.1.1.7 (cluster 1)	Mar-Apr 2021	UK	54	Yes		
B.1.1.7 (cluster 2)	May-Jun 2021	USA	67	Yes		
**Delta (B.1.617.2)**						*Delta overall: rises from 0 to 0.05% before Omicron (5% adv.)*
AY.9.2 (cluster 1)	Sep-Nov 2021	Slovakia	135	Yes		
AY.103 (cluster 2)	Oct-Dec 2021	USA	124	Yes		
AY.4 (cluster 3)	Aug-Dec 2021	UK	179		possibly linked w/cluster 4	
AY.4 (cluster 4)	Jul-Dec 2021	UK	599	Yes		

ORF3a.IORF1 minTRS-B — *nodes with ≥30 descendant genomes are listed*

Taxonium mutations: A26115C
CoV-Spectrum query: LINEAGE* & A26115C — *≥5 independent emegence events*
Amino acid mutations: E241D

Variant / Lineage	Approx. Date	Location Focus	N=	Indep?	Links	Notes
**B.1.1.222**	Dec-May 2020-1	Mexico, USA	45	Yes		
**Alpha (B.1.1.7)**						
B.1.1.7 (cluster 1)	Feb-May 2021	Germany	101	Yes		*plus 23 additional German sequences nearby on tree in B.1.1.7*
B.1.1.7 (cluster 2)	Mar-May 2021	Romania, Europe	46	Yes		
B.1.1.7 (cluster 3)	Mar-May 2021	USA	302	Yes		
**Delta (B.1.617.2)**						
AY.122 (cluster 1)	Aug-Dec 2021	Europe	76	Yes		

ORF3a.IORF2 minTRS-B — *nodes with ≥30 descendant genomes are listed*

Taxonium mutations: T26187C
CoV-Spectrum query: LINEAGE* & T26187C — *≥8 independent emegence events*
Amino acid mutations: D265D

Variant / Lineage	Approx. Date	Location Focus	N=	Indep?	Links	Notes
**B.1.501**	Jun-Jul 2020	USA	45	Yes		
**Alpha (B.1.1.7)**	Feb-May 2021	Germany, Denmark	246	Yes		
**Delta (B.1.617.2)**						
AY.98 (cluster 1)	Oct-Dec 2021	UK	196	Yes		
AY.103 (cluster 2)	Nov-Dec 2021	USA	40	Yes		
AY.100 (cluster 3)	Sep-Dec 2021	USA	69	Yes		
AY.3 (cluster 4)	Sep-Dec 2021	USA	39	Yes		
AY.4 (cluster 5)	Nov-Dec 2021	UK	193	Yes		
AY.39.1 (cluster 6)	Jul-Dec 2021	Australia	5166	Yes		*32% all Aussie Delta seqs; 0% rising to 100% of all sequences in lineage (6-10% adv)*

Table S1. Convergent evolution of TRS-B and minTRS-B sites within SARS-CoV-2. Identified nodes on a phylogenetic reconstruction of SARS-CoV-2 evolution in humans representing clusters of novel TRS and sgmRNA emergence (see Methods). For each emergence event, the parental lineage, approximate date ("Approx. Date") and location ("Location Focus") of circulation are annotated, as well as number of identified descendant genomes within each cluster ("N="), whether it appears to be a true independent emergence event ("Indep?", see Methods) and whether it has further extended homology to the 5′UTR ("+Homol?"). Each cluster is further annotated with potential interrelationships ("Links") and additional notes, including context to its spread based on analysis using CoV-Spectrum (29).
