# Supplementary material for: Emergence of SARS-CoV-2 subgenomic RNAs that enhance viral fitness and immune evasion
Source: PLoS Biol. 2025 Jan 21;23(1):e3002982. doi: 10.1371/journal.pbio.3002982 (PMC11774490; doi:10.1371/journal.pbio.3002982)
Supplement: Attachment — Submitted filename: 240628_response-to-reviewers-comments.pdf [file pbio.3002982.s017.pdf]

Response to Reviewers for

Emergence of new subgenomic mRNAs in SARS-CoV-2

Reviewer #1:

Overall, the manuscript is well written and describes some interesting data consistent with adaptation of SARS-CoV-2 to humans. Work on SARS-CoV-2 will naturally be of interest to a fairly broad audience and data describing how the virus is changing in response to infection of humans is useful for surveillance, vaccine development and also the study of basic virology. However, some data is lacking to fully support the conclusions drawn and discussion of the findings in the context of the wider CoV field, outside SARS-CoV-2, is sparse.

> **We thank the reviewer for their comments — as detailed below and in the revised manuscript, we have now generated data using a series of reverse genetics mutants that provide more direct support for conclusions of this work. We have also expanded our discussion in line with the reviewers' suggestions; we agree the manuscript is strengthened by this addition.**

Specific points:

* The coding capacity of CoVs is increasingly being recognized to be significantly larger than the historically annotated genes. Early work using Northern blot to detect viral transcripts often produced numerous bands in addition to the expected sgRNAs. These were frequently written off as non-specific background bands due to a lack of canonical TRSs needed to generate the additional sgRNAs. However, increasingly, additional transcripts are being described for several different CoVs that must use alternate mechanisms for transcription. The data presented in this work does not rule out the possibility that the N.iORF3 sgRNA is expressed from a minimal, non-canonical TRS in parental strains and mutation to a canonical TRS allows for increased expression, and therefore detection, of the sgRNA. By similarity, this would also apply to other sgRNAs described in the work. Although a canonical TRS will likely give maximum expression of an sgRNA from a given location in the genome, functional non-canonical TRSs have been reported. Including, as acknowledged by the authors, a shorter TRS for the SARS-CoV-2 E sgRNA. Although the N.iORF3 sgRNA could not be detected in EU1 infected cells by end-point or qRT-PCR, evidence of this transcript was shown in supp Fig2b using nanopore sequencing of end point PCR products. This was at a lower level than that seen in Alpha infected cells but that should be expected if a non-canonical TRS is being used. This definitely warrants a fuller description in the text and further investigation.

> **We have included a more explicit discussion of the low level expression of N.iORF3 sgmRNA by viruses which do not contain the TRS-B mutations (lines 391-397).**
>
> **The reviewer is correct that there is low expression with the wild-type sequence (visible with quantification by sgmRNA-specific RT-qPCR in Figure S5C). However, it must be emphasised that there is a "baseline" level of non-canonical sgmRNAs produced <u>throughout</u> the SARS-CoV-2 genome during infection, often generated at sites which bear some resemblance to the**

canonical TRS-B. Without mutation to canonical TRS sequence, there is nothing remarkable about the N.iORF3 site and its transcription: the N.iORF3 sgmRNA is within “background” levels of non-specific sgmRNA generation in ancestral isolates. As an illustrative example for the reviewer, data profiling the transcriptional response of SARS-CoV-2 using next generation sequencing shows that in the absence of the novel TRS-B emergence, generation of sgmRNA does occur at this position along with many, many others — it is very much in the middle of the “noise” of background non-specific sgmRNA generation across thousands of sites. A histogram (log-scale) of abundance is shown below.

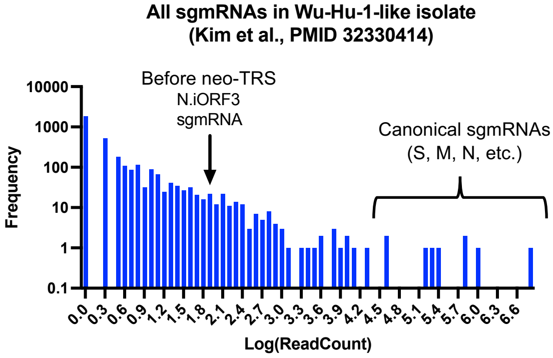

These findings are also in line with previous literature from TGEV genetics experiments, which we have now included in the discussion.

Furthermore, western blot data in Fig 3b clearly shows a band corresponding to N.iORF3 in lanes B and Beta, although these should not express the additional sgRNA.

We discuss the N.iORF3 band in Beta and a potential reason why this might appear (i.e. proteolytic cleavage) despite the absence of the N.iORF3 TRS (lines 239-246).

I don't think the possibility that the sgRNA is expressed from a non-canonical TRS and this is mutating to produce a canonical TRS weakens the findings of the work. The virus is clearly changing upon continued replication in humans, including upregulating expression of one or more sgRNA(s). In addition, data describing additional mechanisms by which CoVs increase their coding capacity and around the precise requirements for production of sgRNAs is a fascinating and an understudied area that needs more attention.

We (of course) agree this is a fascinating area! The discussion now elaborates more on the coding capacity and plasticity of CoVs (lines 370-387).

* How common are AUGs within kozak consensus upstream of a plausible ORF throughout the genome? For this to be maintained within the virus suggests that there may be an existing mechanism for expression.

We investigated ways in which we could correlate neo-TRSs with downstream AUGs in good Kozak context in a way that would be statistically meaningful, however we found this to be an impossible task — especially given the rather expansive list of “plausible” ORFs that must be considered given RiboSeq data showing translation of short and non-canonical ORFs (PMID: 32906143).

* It is obvious that as yet not fully understood elements beyond the TRS itself play a role in regulating TRS usage. Reference to earlier work where additional TRSs have been inserted into a CoV genome to allow generation of a new TRS would be useful (such as work in MHV and IBV). More detailed discussion of TRSs in general would also be beneficial. A significant body of work exists for TGEV but work on MHV and IBV is also relevant and important.

This has now been greatly expanded in the discussion (lines 399-413).

* The possibility that sgRNAs may be functioning at the RNA level rather than as mRNAs is not discussed at all.

We now discuss more explicitly how the transcriptional programme in N.iORF3-expressing viruses is comparable to WT virus — this is in contrast to earlier in other nidoviruses where the introduction of a new sgmRNA at the 3' end of the genome results in a knock-on effect on the transcriptional programme of other sgmRNA expression. We agree that this aspect is worth highlighting.

Reviewer #2:

In this manuscript, Mears et. al. examine novel sub genomic RNAs (sgRNAs) produced during SARS-CoV-2 infection. The authors focus their efforts on the characterization of one novel sgRNA created by the R203K, G204R mutation, which is present in the Alpha, Gamma, and Omicron variants of concern. At the nucleotide level, the R203K, G204R mutation creates a novel Transcription Regulatory Sequence (TRS), resulting in the production of a sgRNA encoding N.iORF3, a putative protein consisting amino of acids 210-419 of SARS-CoV-2 Nucleocapsid. Mears et al. also provide some evidence that other novel sgRNAs may be present in circulating SARS-CoV-2, the significance of which merit further study.

While this reviewer will refrain from commenting on the phylogenetic/bioinformatics portion of this study, there are unfortunately several major issues with the rest of the manuscript as written. First, the authors explicitly brush aside other studies examining both the R203K, G204R and analogous mutations (R203M in delta) at the protein level, thus failing to put their own results into its proper context. From a technical/experimental standpoint, several inadequacies are also present in the author's characterization of N.iORF3. While data is presented confirming a novel sgRNA is being produced, evidence that it is translated into a novel protein is weak. Furthermore, the authors attempt to ascribe a role for N.iORF3 in inhibition of the IFN response, yet do not provide sufficient evidence that this would be true during SARS-CoV-2 infection. Finally, the presence of this novel sgRNA has already been reported by other studies, weakening the novelty of this manuscript given the previously mentioned shortcomings regarding characterization.

We thank the reviewer for their constructive review. Motivated by their suggestions, we have now generated a series of virus mutants by reverse genetics that have allowed us to dissect the contributions of amino acid changes in N vs. the production of N.iORF3 — and to assess relative fitness of these viruses.

Major Issues:

Issue 1:

Line 6 - 7: "While the latter mutations (R203K, G204R) appear unremarkable at the protein level." and lines 32-33: "R203K,G204R mutations appear unremarkable on an amino acid level."

These statements are completely discordant with the current literature, which strongly suggest that the R203K, G204R mutation is absolutely having an effect on the protein level. Specifically, Johnson et al. (PMID: 35728038) provides data suggesting the R203K, G204R mutation alters the phosphorylation of nucleocapsid during infection of Vero and Calu3 cells, Wu et al. (PMID: 34822776) proposes a structural model by which the mutation may alter nucleocapsid's binding to RNA, and Zhao et. al. (PMID: 33837182) demonstrate that R203K, G204R modulates phase separation of the nucleocapsid protein. The authors should discuss why these past studies are wrong, discuss the introduction of a TRS site in the context of these other functions, or else remove such a strong and incongruent statement from the abstract and main text.

> **We apologise for generating the impression that the amino acid changes might not be relevant, our point was that *prima facie* the conservation of charge and sequence in this linker region would appear unremarkable. We agree that this could be misleading, so have removed this statement and now discuss these roles extensively (lines 326-336).**
>
> **We would emphasise to the reviewer that <u>none</u> of the above papers they cite recognise that the mutations they have studied also generate a TRS site and a novel subgenomic RNA species.**
>
> **To this end, we have now carried out carefully-controlled reverse genetics experiments to dissect the amino acid vs. TRS-B contributions. When we knock out the N.iORF3 TRS but preserve the amino acid sequence, we find distinct phenotypes when compared to viruses in which the amino acid sequence has been mutated (lines 260-287, Fig 5 and S9).**
>
> **This adds a new dimension to our understanding of the functional implications of the N:R203K,G204R mutations – our data support a model in which <u>both</u> the amino acid and the nucleotide level changes contribute to viral fitness — and we thank the reviewer for motivating us to examine this further.**

Issue 2:

Line 103 -104 and Figure 3b and 3c: "we also observed 103 a band at ~25 kDa in B.1.1 and Alpha-infected cells (Fig. 3b), consistent with the mobility of 104 recombinant N.iORF3 protein (Fig. 3c)"

If the authors wish to suggest In Figure 3b that the lower molecular weight bands are consistent in size to recombinant N.ORF3, then recombinant N.ORF3 should be ran on the same gel. Referring to a different figure (Figure 3c) is not sufficient. Additionally, at least one study suggests that nucleocapsid undergoes extensive proteolytic cleavage (PMID: 34548480). The authors need to discuss this as an alternative explanation for their results, especially given the numerous other bands of equal or greater intensity throughout the gel. Finally, by this reviewer's eye a faint N.iORF3 band seems to be present in the Beta variant,

despite it lacking the R203K, G204R mutation. This band's presence in the Beta variant is in direct conflict with the authors' own conclusion: that this band represents the N.iORF3 protein is being translated from a novel sgRNA produced by the R203K, G204R mutation. More evidence that N.iORF3 is actually being expressed a detectable levels is needed.

More western blots are now included to provide a clearer view of the band which corresponds to N.iORF3 (Fig 4E and S8) and the possibility of proteolytic cleavage is discussed, particularly with regard to the band which is visible in Beta infection (Lines 236-246).

We additionally perform western blots with mutant Alpha-backbone viruses in which the N.iORF3 TRS is either silently mutated or reverted back to WT (i.e. NR203,G204). In both cases, the putative N.iORF3 protein band disappears, which we think is compelling evidence of its identity (Fig 5B and S8C).

Issue 3:

Lines 104 - 113, and Figure 3c and 3d: The authors own data suggests that N.iORF3 is expressed at low levels relative to full length N (RNA - Extended Data 1d-e, Protein - Figure 3b). Additionally, SARS-CoV-2 infection is known to be more sensitive to IFN compared to SARS-CoV-1 (PMID: 32938761). With this in mind, merely showing a modest effect on ISG induction when overexpressing N.iORF3 and poly-ic in HEK293 cells is unconvincing given its artificiality. At minimum, the authors need to perform additional experiments showing that the R203K, G204R mutation provides resistance to IFN relative to wildtype during SARS-CoV-2 infection. Ideally, the authors would also link this resistance to the presence of N.iORF3.

We have now performed experiments with reverse-genetics derived viruses in IFN-competent cells and found a phenotype when the N.iORF3 TRS is mutated to prevent its expression mutated (lines 260-287, Fig 5 and S9). This result indicates a role in viral fitness, for which IFN antagonism is one possible mechanism. We believe unpicking the exact role of N.iORF3 in IFN antagonism and other processes in viral infection will warrant a larger further study and this is discussed in the text (lines 353-368).

Issue 4:

Discussion: Related to issue 1 above, several analogous mutations at this locus SARS-CoV-2 nucleocapsid, including the R203M mutation in delta and T205I mutation in beta (see authors own Figure 1C). These mutations have dominated individual lineages (and the total circulating SARS-CoV-2 in general in the case of R203M/Delta) at various points in the pandemic, yet they do not create novel sub genomic transcripts. While this does not preclude a novel sgRNA and N.iORF3 contributing to the (previously reported) enhanced fitness of the R203K, G204R mutation, it implies that it may not be necessary and strongly suggested is isn't the sole contributor. The authors need to address why these other mutations emerged and are alone sufficient to enhance replication/fitness without producing novel sgRNA (PMID: 34735219).

We agree with the reviewer fully — this has now been comprehensively addressed by experiments with reverse genetics-generated mutants that confirm the sgmRNA itself (and not just the amino acid changes) contribute to

viral fitness. We have expanded our discussion accordingly. Our aim in this manuscript is to describe an underappreciated phenomenon: the emergence of novel TRS sites and sgmRNAs — rather than focus on the amino acid changes in the linker region. In line with the suggestions from the academic editor below, have rearrange our manuscript as well to ensure this focus remains prioritised.

Issue 5:

Novelty: Other studies have already been published suggesting a new sgRNA is produced by the R203K, G204R mutation (PMID: 34541432). Given the other shortcomings, it is hard to see how merely confirming the presence of this transcript with a different methodology (RT-PCR/public data sets vs deep sequencing of clinical samples) provides any new information. The authors should focus on strengthening the other aspects of their manuscript to increase its novelty.

Following the reviewers' suggestion, we have significantly strengthened the manuscript, most notably through new reverse genetics experiments in which the TRS-B has been silently mutated. We note that none of these mutants, which allow us to deconvolve amino acid vs. TRS-B/sgmRNA contributions to virus fitness have been described in the literature.

The work cited by the reviewer is an observational study that repurposes ARTIC amplicon sequencing data from SARS-CoV-2 surveillance. It does not accurately estimate abundance of the RNA, and does not attempt to carry out any functional molecular biology or virology to understand this sgmRNA or its role.

We believe our work here (following the suggestions from the reviewers), which explores the phenomenon of neo-TRS and sgmRNA emergence, as well as the functional role of the most prominent TRS greatly add to the novelty and scientific impact of this study.

Minor Issues:

Issue 6:

Line 72/ Figure 2b: The clarity of these gels would increase significantly if the ladder was labeled base with pair markers.

The gels (Fig 2F and 3E) have now been annotated with bp markers.

Issue 7:

Line 87 - 97: While the authors RT-qPCR assays do indicate a N.iORF3 sub-genomic assay is present at the RNA level with ~1% the expression of full length N, this review wonders if a Northern Blot would not be more convincing.

We cordially disagree with the reviewer that a northern blot would be more convincing than next-generation sequencing and a thoroughly calibrated and validated RT-qPCR TaqMan assay (see Fig S5).

COMMENTS FROM THE ACADEMIC EDITOR:

My biggest critique is that the current organization is a bit awkward. They begin with a very narrow focus on N.iORF3, then they present a broader search for other similar mutations and wrap up by describing some of the other variants that are common. It seems to me that the flow would be improved if they began with their broad search for new TRS-B sites (ED-Fig 4a), then continued with further characterization of the three most common variants they observed. I think the overall content would mostly be unchanged, but the flow would be more natural.

We thank the editor for this suggestion and have rewritten the manuscript accordingly. We agree the flow is much more natural.

Another aspect that I found a bit confusing was why they primarily refer to these substitutions by their AA-level designations when it is really the nucleotide-level substitutions that are important for their findings. For example, they talk about "R203K, G204R" as a double substitution. However, there are really three nt-level substitutions that underly these AA-level changes. I understand that the community has been much more focused on the AA-level changes, but I would encourage the authors to embrace the nt-level designations as the primary level of description in their paper.

We have amended the text and figures to principally refer to the nucleotide mutations.

Additional comments:

1. Fig. 1a: the legend says that the lineage defining mutation for B.1 is indicated, but it's not.

This has been amended.

2. Fig. 1a: I believe the "K204R" reference in the legend is a typo.

This has been amended.

3. The authors need to explain why N.iORF3 expression was compared against E expression (Fig. 2d).

N is very highly expressed and E is relatively poorly expressed, so these targets provide relevant comparators to understand N.iORF3 expression in the context of canonical sgmRNA expression. This is now explained more explicitly in the text (lines 200-203).

4. Fig. 2b legend should include a description for all of the labels.

The legend has been amended to describe gel labels.

5. Manuscript needs a better description of why Flu-A NS1 is an appropriate positive control.

A fuller explanation of the choice of NS1 as a positive control is now included (line 295).

6. The manuscript needs more context for the analysis shown in Fig. 3d. Yes, N.iORF3 can act as an innate immune signaling antagonist, BUT it's activity seems to be lower than that of full-length N and it's expressed at a much lower level. So, would this activity really be expected to play a substantial role during infection?

We now discuss the activity of N.iORF3 more in the discussion, including other reports which have shown distinct roles in IFN antagonism and other viral processes. We suggest it is one potential explanation for the attenuation we see in our mutant viruses (lines 353-363). The exact mechanism of N.iORF3 (especially at its low expression level) will be the subject of future studies.

7. Lines 20 and 22, refs should be provided for the statements about the frequencies of these different mutations.

All references to mutation frequencies in the text are now accompanied by references to GISAID, CoV-Spectrum or Nextstrain, as appropriate.

8. Lines 84-85: rephrase "but not from the B.1 lineage." Isn't B.1.1 also part of the B.1 lineage?

This has now been rephrased to “non-B.1.1 lineages” (line 121).
