# Supplementary material for: Emergence of SARS-CoV-2 subgenomic RNAs that enhance viral fitness and immune evasion
Source: PLoS Biol. 2025 Jan 21;23(1):e3002982. doi: 10.1371/journal.pbio.3002982 (PMC11774490; doi:10.1371/journal.pbio.3002982)
Supplement: Attachment — Submitted filename: 241011 Response to Reviewers.pdf [file pbio.3002982.s018.pdf]

Response to Reviewers — 14 August 2024

Emergence of new subgenomic mRNAs in SARS-CoV-2

Reviewer #1:

The updated manuscript addresses the queries raised in the last review providing a more comprehensive analysis of control of expression of the novel sgmRNA and a more detailed discussion in the context of previous knowledge. There are still some outstanding comments/questions and the discussion could still be expanded a little:

> **We thank the reviewer for their constructive comments — we have performed additional experiments and expanded the discussion to comprehensively address all outstanding points raised by the reviewers during this second round of review. We think these suggestions have greatly strengthened the manuscript.**

1. Line 62-63: Although the spectrum of sgRNAs identified by this approach may overrepresent what is truly made, it is misleading to gloss over the fact that some viruses make what have only recently been recognised as functional sgmRNAs due to their use of a minimal TRS (and actually quite efficiently). See data on IBV.

> **We have added a sentence in to clarify this point (lines 62-63). Existing data suggests that SARS-CoV-2 is comparatively stringent in its TRS usage, and we agree this is important general feature to clarify in contrast to other CoVs.**

2. Fig 5. Did you confirm that these mutations abolish sgRNA expression?

> **Yes. We have included additional sgRNA qPCR data to show this abolition in Fig S9 and highlight this fact in the text. (lines 260-264).**

3. Alpha-silTRS - It would be most informative to include a control where the minimal TRS (GCACA) is mutated on its own. And also, there is no confirmation that it is not the different codon usage for the KR that's the problem here.

> **Our aim in this work to provide a +/- TRS set of recombinant viruses. We chose a 'thorough' TRS mutation to ensure if there was a phenotype that we would catch it, since we suspected it might be quite subtle. We agree that this may be interesting to examine "titrating" levels of TRS expression (e.g. by progressively abolishing the core/minimal TRS sequences as suggested here), but this is beyond the scope of this work.**
>
> **For clarity, in case there is confusion: the minimal TRS for SARS-CoV-2 is "ACGAAC" (not GCACA, which is the mutated sequence we chose), as utilised upstream of the Envelope gene and ORF6. In our "silTRS" mutant, we mutate the minimal TRS to "gaGcAC", and not the preceding "AA" that forms the 8nt consensus full TRS. We also mutate 1 nucleotide outside (downstream) of the**

core TRS (U>A) that has homology to the 5' UTR, for which homology is also present downstream of the minimal TRS sequence for the Envelope gene.

With regards to codon usage, we agree we haven't demonstrated that codon usage is not the issue. Naturally, it will not be possible to create silent mutations without some change of codon usage, and we wanted to indicate that our design has taken this into consideration. (line 255 of previous manuscript, now line 257).

4. Fig 5H - why not test this with the RG viruses that you've made to link back to biological relevance? I appreciate that redundancy in viral antagonists of IFN induction/signalling may mask the effect but it's still a valuable experiment to include.

We agree this is a good suggestion – thank you! We have performed an additional set of experiments, presented in Figure 6 and S10, to test the fitness of the RG viruses in cells deficient for cytoplasmic dsRNA sensors, to test whether N.iORF3 does indeed antagonise signalling in infection. We found a modest recovery of Alpha-silTRS replication in the absence of RIG-I, indicating of a role in RIG-I antagonism (described in lines 295-319 and discussed in lines 382-404).

5. 316-7 - I strongly query the legitimacy of calling this a "novel" sgmRNA specific assay. This has been done for other CoVs in the past.

We have changed "novel" to "custom" in line 343 to more accurately reflect that our assay was developed and validated for the purposes of this study, not that it is a novel concept.

6. Lines 396-397 - I think it worth expanding the discussion here. It has also been found that a minimal TRS is actually more efficient/preferentially used over a new inserted complete TRS. There may be differences in how much flexibility is tolerated between the CoV genera.

We agree this area warranted more discussion. An additional paragraph has been included to give more context to TRS usage and the variety of transcription strategies in CoVs and Nidoviruses in general (lines 443-455).

7. Lines 407-409 - Isn't this surprising though? Would you propose then that the overall transcriptional output increases to keep the other sgRNA levels the same AND include expression of this new sgRNA? Maybe comparing additional sgRNA levels would be useful here?

We agree that the acquisition of a new sgmRNA with minimal perturbation of the overall transcriptional programme is somewhat surprising considering work with other Nidoviruses (e.g. Snijder lab findings with Equine Arterivirus). On the other hand, it is perhaps less surprising given the apparent complexity of SARS-CoV-2 sgmRNA abundance apparent in RNAseq data since the start of the pandemic (which doesn't follow a gradient of abundance from 3' to 5'), and the apparent lack of significant changes in the transcriptional programme when variants such as Omicron BA.5 & descendants have naturally abolished

the ORF8 TRS-B sequence (see, e.g. https://virological.org/t/repeated-loss-of-orf8-expression-in-circulating-sars-cov-2-lineages/931).

When examining sgmRNA expression in our mutants in response to point 2 above, we did notice some small differences in E expression in our mutants – we speculate in the discussion (lines 470-475) that there are other mutations in Alpha and other B.1.1-lineage viruses which accommodate the acquisition of a new TRS-B and rebalance the transcriptional programme.

With practical constraints on qPCR probe design, it was not possible to examine other sgmRNAs due to the repetitive and AU-rich nature of the regions downstream of their TRS-Bs, but we agree examining the overall sgRNA transcriptome would be valuable for future work (provided they are not subject to the biases we have discussed in the paper already).

Reviewer #2:

This manuscript form Mears et al. details the how mutations among SARS-CoV-2 variants lead to expression of novel sub-genomic transcripts via the creation of new transcription regulatory sequences (TRSs). While evidence from publicly available datasets is examined demonstrating the frequency at which these novel TRSs are created, the authors focus their efforts on the characterization of two: one within Nucleocapsid created through the R203K, G204R substitution (creating a novel N.iORF3) and a second within ORF1ab (creating a novel nsp16.iORF1). Data is presented demonstrating the expression of these novel transcripts at the mRNA and protein levels among clinical isolates, viral stocks, and viruses created using reverse genetics.

Compared to the authors initial submission, this version of the manuscript is greatly improved.

We thank the reviewer for their positive assessment.

Summarizing the authors' specific claims:

Major Points with strong supporting evidence:
* Creation of novel TRS sites are not isolated events (Fig. 2A)
* The novel N.iORF3 is expressed at the RNA (Fig. 2F, Fig. 4A-D)) and protein (Fig. 4E) levels
* The protein band corresponding with N.iORF3 is specifically caused by the R203K, G204R mutation (Fig. 5B)
* N.iORF3 expression enhances viral replication and fitness, and its removal of the TRS by reverse genetics attenuates the Alpha variant even while maintaining the mutation on the amino acid level (Fig. 5C-E, Fig. S9),
* Nsp16.iORF1 is expressed at the RNA level (Fig. 3E).

Overall, these 5 points are very well supported both by the figures cited above as well as the supplemental material. However, the authors attempt to make a sixth claim regarding the function of N.iORF3 in inhibiting IFN signaling, which I still believe lacks sufficient evidence. This point is elaborated on below.

We agree with the reviewer that this functional demonstration of N.iORF3 is an important addition — we have now included additional experiments examining our RG viruses in the absence of cytoplasmic dsRNA sensors, which show N.iORF3 antagonises the innate immune response. (Details discussed below, Figs. 6 and S11).

Major Weaknesses

* Presented data inadequately support the claim the N.iORF3 functions to inhibit Type I IFN signaling.

While N.iORF3 is convincingly playing some role in viral replication and fitness (Fig. 5C-E, Fig. S9), I remain unconvinced that N.iORF3 functions to inhibit IFN signaling during infection. To support this claim, the authors ectopically express SARS2 N and SARS2 N.iORF3 in 293T cells, transfect poly:iC, and measure the induction of IFNB and IFTIT mRNA (Fig. 5F-H). I have several lingering critiques of this experiment, all of which I stated in my initial review. First, according to the figure legend and the western blot data in Fig. 5H, SARS2 N and SARS2 N.iORF3 are being expressed at equal amounts. However, at each concentration N.iORF3 appears to less effectively inhibit IFNB expression relative to full length SARS2 N (though the authors do not directly compare the two with statistics). However, the authors own data suggests that N.iORF3 expression is approximately 100-fold lower than full length SARS 2N (Fig. 4D) during infection. Taken together, these data may me skeptical that N.iORF3's primary role is the inhibition of Type I IFN. To summarize succinctly, while the data in Fig. 5H suggests that ectopic expression of N.iORF3 can inhibit IFN, it may have less of an effect compared full length SARS2 N, and due to lower levels of expression it seems unlikely that it would have this effect during infection.

A relatively straightforward experiment can be performed to address this issue. If N.iORF3's major role is the inhibition of interferon signaling, then a virus expressing N.iORF3 should be more resistant to interferon treatment relative to one that does not. Fortunately, the authors already have all the tools needed to test this. I would suggest that the Alpha-WT, Alpha N:RG, and Alph-siTRS be used to infect Vero-E6 TMPRSS2 cells with and without IFN pretreatment. If the authors' current hypothesis is correct, that Alpha-N:RG and Alpha-siTRS should be more sensitive to Interferon treatment when compared with Alpha-WT. For an example of this sort of assay, see Fig. 1 of Lokugamage et al. (PMID: 32938761). If not, then it is likely that N.iORF3 has a different function. This is a fairly easy experiment to perform and would greatly strengthen the authors assertion that N.iORF3 functions to inhibit interferon signaling during infection.

We agree fully that a direct demonstration of the advantage N.iORF3 provides in suppressing interferon signalling would strengthen the manuscript — we have now performed an additional set of experiments, presented in Figures 6 and S10, which examine the growth of our RG viruses in cells lacking the cytoplasmic dsRNA sensors RIG-I and MDA5. We opted to knockout sensors at the start of the innate immune signalling cascade, rather than stimulating with IFNa, to test the hypothesis that N.iORF3 antagonises IFN induction.

We describe that while Alpha-WT replicates slightly better in RIG-I knockout cells, Alpha-silTRS replicates significantly better, supporting a role in antagonising RIG-I sensing.

Interestingly, the phenotype of the silTRS virus is not entirely restored to WT levels, especially regarding the kinetics of virus growth which remain delayed. We discuss the various described and potential roles for N.iORF3, particularly the potential for a role in packaging and speculate about how N.iORF3 may be able to perform such roles at low expression levels, while emphasising the need for more work in this area to explore the complexity in the future.
